# The RNA receptor RIG-I binding synthetic oligodeoxynucleotide promotes pneumonia survival

**DOI:** 10.1172/jci.insight.180584

**Published:** 2024-11-08

**Authors:** Yongxing Wang, Vikram V. Kulkarni, Jezreel PantaleónGarcía, Michael K. Longmire, Mathilde Lethier, Stephen Cusack, Scott E. Evans

**Affiliations:** 1 Department of Pulmonary Medicine, The University of Texas MD Anderson Cancer Center, Houston, Texas, USA.; 2 The University of Texas MD Anderson Cancer Center UTHealth Houston Graduate School of Biomedical Sciences, UTHealth Houston, Houston, Texas, USA.; 3 European Molecular Biology Laboratory, Grenoble, France.

**Keywords:** Cell biology, Immunology, Bacterial infections, Influenza, Innate immunity

## Abstract

Pneumonia is a worldwide threat to public health, demanding novel preventative and therapeutic strategies. The lung epithelium is a critical environmental interface that functions as a physical barrier to pathogen invasion while also actively sensing and responding to pathogens. We have reported that stimulating lung epithelial cells with a combination therapeutic consisting of a diacylated lipopeptide and a synthetic CpG oligodeoxynucleotide (ODN) induces synergistic pneumonia protection against a wide range of pathogens. We report here that mice deficient in TLR9, the previously described receptor for ODN, still displayed partial ODN-induced protection. This prompted us to seek an alternate ODN receptor, and we discovered by mass spectroscopy that the RNA sensor RIG-I could also bind DNA-like ODN. ODN binding by RIG-I resulted in MAVS-dependent pneumonia-protective signaling events. While RIG-I is essential to native defenses against viral infections, we report that therapeutic RIG-I activation with ODN promoted pathogen killing and host survival following both viral and bacterial challenges. These data indicate that maximal ODN-induced pneumonia protection requires activation of both the TLR9/MyD88 and RIG-I/MAVS signaling pathways. These findings not only identify what we believe to be a novel pattern recognition receptor for DNA-like molecules, but reveal a potential therapeutic strategy to protect susceptible individuals against lethal pneumonias during periods of peak vulnerability.

## Abstract 2

Although known to sense RNA, we find that RIG-I can also be therapeutically targeted with DNA-like molecules to induce protection against viral and bacterial pneumonias.

## Introduction

Pneumonia has long been recognized as a leading cause of death among healthy and immunocompromised people worldwide. Novel strategies are urgently needed to protect patients with impaired immunity against lethal pneumonias caused by emerging respiratory pathogens like SARS-CoV-2 and antibiotic-resistant nosocomial pathogens like methicillin-resistant *Staphylococcus aureus* (1–3).

Innate and intrinsic immune responses protect the host by recognizing broad classes of pathogens, detecting common pathogen-associated molecular patterns (PAMPs) by germline-encoded pattern recognition receptors (PRRs). Activation of evolutionarily conserved PRRs rapidly promotes signaling and antimicrobial effector events that protect the host against invading pathogens without direct reliance on immunologic memory or epitope-level specificity (4, 5). Thus, therapeutic manipulation of PRRs has the potential to improve survival of otherwise lethal lung infections.

Many immunocompromised patient groups, such as patients with hematological malignancies, people living with uncontrolled HIV infections, and recipients of solid organ or hematologic stem cell transplants, have heightened susceptibility to pneumonia owing to quantitative or functional defects of leukocyte-mediated immunity (3). In order to protect these hypersusceptible individuals, our laboratory investigated means to manipulate the innate immune capacities of long-lived, relatively chemotherapy-insensitive lung epithelial cells. In addition to acting as a physical barrier to pathogen invasion, airway and alveolar epithelial cells sense and respond to the presence of pathogens. Upon PAMP detection by lung epithelial PRRs, not only do epithelial cells modulate the lung leukocyte environment through expression of cytokines and chemokines, they also elaborate molecules that are directly microbicidal, including antimicrobial peptides and ROS (6, 7). Leveraging these defensive mechanisms, we identified a pneumonia-protective, synthetic PAMP-based therapeutic comprising a diacylated lipopeptide ligand for TLR2/6 (Pam2CSK4 [Pam2]) and a class C unmethylated cytosine-guanine (CpG) oligodeoxynucleotide ligand for TLR9 (ODN M362 [ODN]. The ODN component is a 25-mer palindromic sequence (5′-TCGTCGTCGTTC:GAACGACGTTGAT-3′) with a complete phosphorothioate backbone (4). A single prophylactic inhaled treatment with this nonintuitive dyad of ligands (Pam2ODN) for these spatially segregated TLRs substantially promotes intrapulmonary pathogen killing and host survival. This phenomenon, termed inducible epithelial resistance, extends to all tested bacterial, fungal, and viral pathogens (8–16).

A striking characteristic of this combination treatment is that the two components act synergistically (14, 17). While either Pam2 or ODN delivered alone yields modest pneumonia protection, delivering the two ligands concurrently affords robust pathogen-killing and survival advantage. Trying to understand how these ligands interact, we previously showed that synergistic protection is entirely lost in TLR2-deficient mice, and we consequently hypothesized that Pam2ODN-induced synergistic protection would similarly be abrogated in TLR9-deficient mice. TLR9 is well-characterized as a primary intracellular sensor for unmethylated CpG motifs, such as those found in bacterial or viral DNA and is established as a principal therapeutic target for CpG-containing ODNs (18). TLR9 is also widely expressed in the lung, including by lung epithelial cells and leukocytes (19), making it a plausible target for our inhaled Pam2ODN therapeutic. However, we report here that TLR9 is not the only receptor for ODN. We found that the RNA sensor RIG-I can also bind DNA-like ODN and that ODN-induced RIG-I activation results in mitochondrial antiviral-signaling protein–dependent (MAVS-dependent) antimicrobial responses. As such, full ODN-induced protection requires activation of both the TLR9/MyD88 and RIG-I/MAVS pathways.

## Results

### TLR-9–independent ODN-induced pneumonia protection.

**Figure 1:**
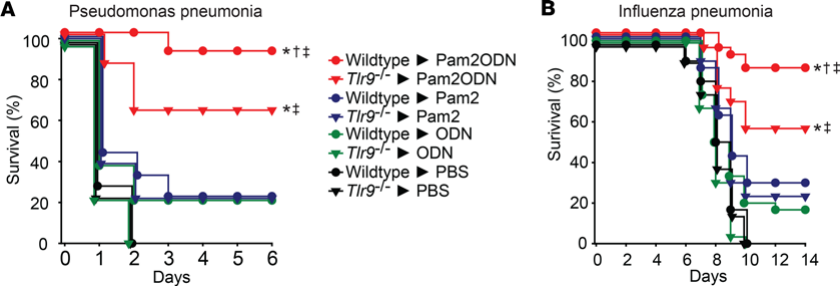
TLR-9 independent ODN-induced protection against pneumonia. (**A** and **B**) Wild-type or *Tlr9^–/–^* mice were challenged with lethal doses of (**A**) *P*. *aeruginosa* or (**B**) influenza A virus 24 hours after nebulized treatment with PBS, Pam2, ODN, or Pam2ODN. *P < 0.01 vs. PBS-treated syngeneic mice, †*P* < 0.05 vs. Pam2ODN-treated *Tlr9*^–/–^ mice, ‡*P* < 0.01 vs. Pam2-treated syngeneic mice, all by log-rank test (Mantel-Cox method).

To test the role of TLR9 in Pam2ODN-induced resistance, wild-type or *Tlr9^–/–^* mice received a single aerosolized treatment of PBS, Pam2, ODN, or Pam2ODN 24 hours prior to challenge with lethal inocula of *Pseudomonas aeruginosa* or influenza A virus (Figure 1). Consistent with our prior observations (14, 16), Pam2ODN profoundly protected the wild-type mice against pneumonia-related death, while treatment with the individual ligands afforded modest, if any, survival advantage. As expected, ODN treatment alone failed to protect *Tlr9^–/–^* mice. However, we were surprised to observe that Pam2ODN treatment led to significantly greater protection than Pam2 alone in the TLR9-deficient mice. This indicated a TLR9-independent ODN-induced mechanism contributing to inducible resistance, prompting us to investigate alternate ODN sensors. Data for all group sizes and comparisons are provided in Supplemental Table 1 (supplemental material available online with this article; https://doi.org/10.1172/jci.insight.180584DS1).

### Identification of RIG-I as an ODN binding partner.

Native and synthetic CpG motif–containing DNA-like molecules are internalized by clathrin-dependent endocytosis and activate endosomal TLR9, initiating antimicrobial signaling cascades (4). Activated TLR9 is retained in endocytic vesicles. However, internalized synthetic CpG ODNs also localize to the cytosol in punctate distribution patterns (20), raising the possibility of a contributing cytosolic ODN receptor. Cyclic GMP–AMP synthase–stimulator of interferon genes (cGAS-STING) and interferon-activated gene *Ifi204* (orthologous to IFI16 in humans) are TLR9-independent detectors of cytosolic foreign DNA (21), so we tested whether these molecules might be an extra-endosomal mediators of ODN-induced protection. As expected, cGAS-, STING-, and Ifi204-deficient mice each demonstrated increased baseline susceptibility to influenza challenges, but the magnitude of Pam2ODN-induced protection against viral or bacterial challenges was unaffected in knockout mice (Supplemental Figure 1), effectively excluding cGAS-STING or Ifi204 as critical to Pam2ODN-inducible resistance.

**Figure 2:**
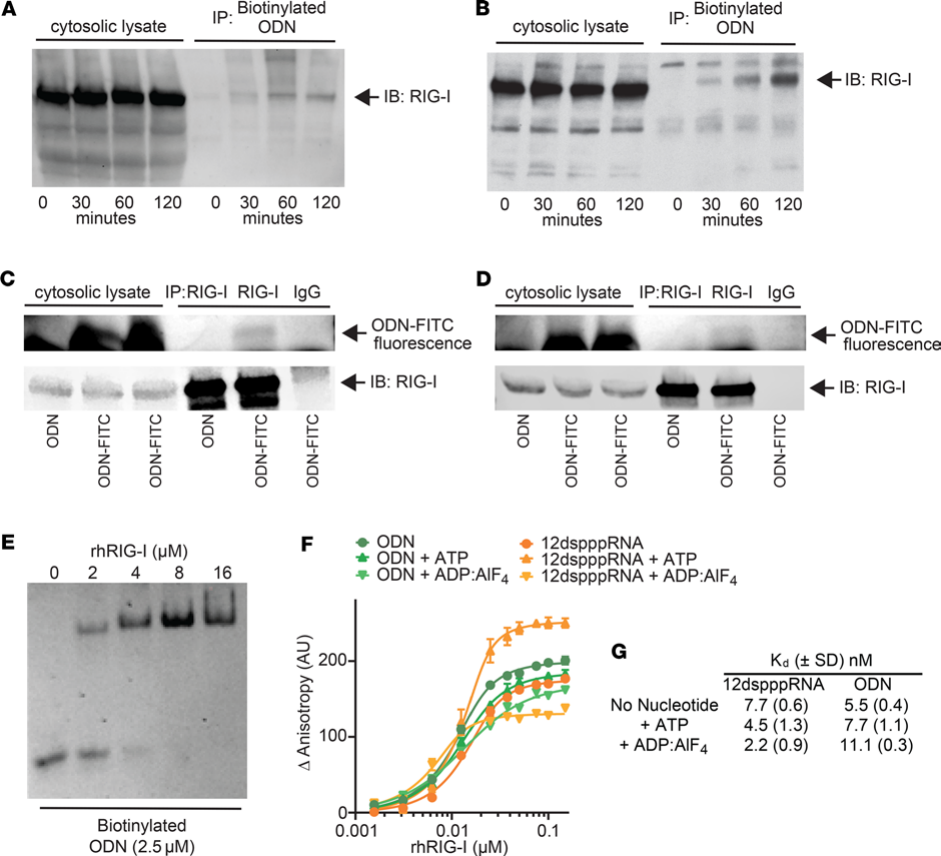
RIG-I is a cytosolic receptor for ODN. (**A** and **B**) Cytosolic lysates from biotinylated-ODN-treated (**A**) human HBEC3-KT or (**B**) murine MLE-15 lung epithelial cells were streptavidin precipitated and probed with anti–RIG-I antibody. (**C** and **D**) Cytosolic lysates from FITC-labeled ODN-treated (**C**) HBEC3-KT or (**D**) MLE-15 cells were immunoprecipitated with anti–RIG-I antibody or IgG isotype control. Fluorescence from FITC-labeled ODN was detected by LI-COR imaging. RIG-I was probed with anti–RIG-I antibody. (**E**) Electrophoresis mobility shift assay of purified rhRIG-I protein incubated with ODN. (**F**) Fluorescence polarization anisotropy of rhRIG-I exposed to ODN or RNA in the presence or absence or ATP or ADP-AlF_4_. (**G**) The equilibrium binding constant (Kd) values of the corresponding fluorescence anisotropy binding curves are shown in **F**. rhRIG-I, recombinant human RIG-I; ADP-AlF_4_, adenosine diphosphate–aluminum fluoride. Data are shown as mean ± SD.

Seeking alternate ODN receptors, human lung epithelial HBEC3-KT cells were treated with ODN or biotinylated ODN. Cell lysates were incubated with streptavidin beads and then the eluted proteins were resolved by PAGE and Coomassie stained. Differentially stained bands were excised and analyzed by liquid chromatography–mass spectrometry (LC-MS), yielding a list of candidate proteins that might function as ODN receptors (Supplemental Figure 2 and Supplemental Table 2). Candidate proteins were validated individually by pull-down assays in biotinylated ODN-treated HBEC3-KT cells and mouse lung epithelial MLE-15 cells. The innate immune receptor retinoic acid–inducible gene I (RIG-I, alternately DExD/H-box helicase 58 [DDX58]) was identified as an ODN binding partner (Figure 2, A and B, and Supplemental Figure 3). To further confirm that RIG-I binds ODN, HBEC3-KT cells and MLE-15 cells were treated with either unlabeled ODN or FITC-labeled ODN, and then the cytosolic lysate was submitted to immunoprecipitation with either anti–RIG-I Ab or IgG isotype control. Using LI-COR imaging, we detected fluorescence from the FITC-labeled ODN in the RIG-I–precipitated samples, both in human and mouse cells, revealing coprecipitation of RIG-I and FITC-labeled ODN (Figure 2, C and D, and Supplemental Figure 3). As in our prior reports (14, 22), this experiment also demonstrates that ODN applied to lung epithelial cells can reach the cytosolic location of RIG-I.

RIG-I, the titular member of the RIG-I–like receptor (RLR) family, is critical to virus detection, sensing short segments of dsRNA and ssRNA with 5′ triphosphate caps (23, 24). Although RIG-I is an RNA receptor, inhibition of RNA polymerase III had no effect on RIG-I interactions with ODN, indicating that RIG-I was not simply binding RNA transcribed from ODN sequences (Supplemental Figure 4) (25, 26). Rather, RIG-I directly interacts with ODN, as demonstrated by electrophoretic mobility shift assay using purified recombinant human RIG-I (rhRIG-I, amino acids 236–925) protein (Figure 2E). Fluorescence polarization anisotropy measures binding constants and kinetics of ligand-receptor reactions (27). In vitro protein binding assays using fluorescence polarization anisotropy demonstrated that the binding affinity of rhRIG-I for ODN is similar to that of rhRIG-I for its native RNA ligand (Figure 2F). However, the addition of ATP or adenosine diphosphate–aluminum fluoride (ADP-AlF_4_) did not impact ODN binding in a manner similar to the effect on RNA binding, suggesting potential differences in the binding mechanics for these two RIG-I ligands (Figure 2, F and G).

### RIG-I is required for maximal ODN-induced pathogen killing and pneumonia survival.

**Figure 3:**
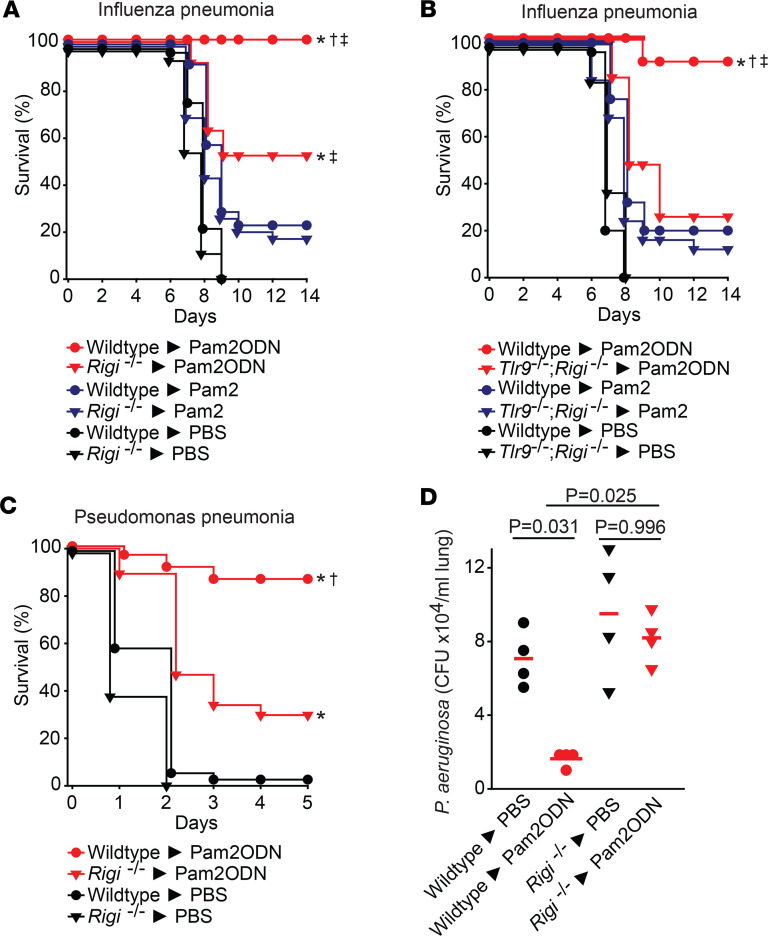
ODN-induced RIG-I activation is required for ODN protection against pneumonia. (**A**) *Rigi^–/–^* or wild-type littermates or (**B**) Wild-type or *Tlr9^–/–^;Rigi^–/–^* double-knockout mouse littermates were challenged with lethal doses of influenza A virus 24 hours after nebulized treatment with PBS, Pam2, or Pam2ODN. (**C**) *Rigi^–/–^* or wild-type littermates were challenged with lethal doses of *P*. *aeruginosa* 24 hours after nebulized treatment with PBS or Pam2ODN. (**D**) Bacterial burden of the lungs in **C** compared by 1-way ANOVA (Kruskal-Wallis method). *P < 0.01 vs. PBS-treated syngeneic mice, †*P* < 0.01 vs. Pam2ODN-treated knockout mice, ‡*P* < 0.01 vs. Pam2-treated syngeneic mice, all by log-rank test (Mantel-Cox method).

To investigate the functional role of RIG-I in ODN-induced protection, *Rigi^–/–^* and wild-type littermates were treated with PBS, Pam2, or Pam2ODN 24 hours before influenza A virus challenge. Whereas Pam2ODN treatment yielded full protection of the RIG-I–replete mice, Pam2ODN treatment of RIG-I–deficient mice afforded protection that was significantly greater than Pam2 alone but significantly less than Pam2ODN treatment of RIG-I–replete mice (Figure 3A). This pattern was strikingly similar to that observed with single ligand versus combination treatment of TLR9-deficient mice, supporting a hypothesis that both TLR9 and RIG-I are required for maximal ODN contributions to Pam2ODN synergistic protection but neither is sufficient alone. Consistent with that hypothesis, *Tlr9^–/–^;Rigi^–/–^* double-knockout mice showed loss of Pam2ODN synergistic protection against influenza A pneumonia (Figure 3B), reflecting impairment of the inducible antiviral response. More surprisingly, we also observed impairment of Pam2ODN-inducible antibacterial responses in *Rigi*-knockout mice. We challenged *Rigi^–/–^* and wild-type littermate mice with *P*. *aeruginosa* 24 hours after inhaled treatment with PBS or Pam2ODN. The survival of Pam2ODN-treated *Rigi^–/–^* mice was significantly worse than that of Pam2ODN-treated wild-type mice (Figure 3C). This observation is congruent with our finding that the bacterial burden of Pam2ODN-treated wild-type mouse lungs immediately after challenge was significantly lower than that of PBS-treated mice, whereas no such inducible difference was achieved in *Rigi^–/–^* lungs (Figure 3D). This unexpected role for RIG-I in inducible antibacterial protection is notable, because, to our knowledge, RIG-I has not been described to participate in inducible antibacterial responses.

**Figure 4:**
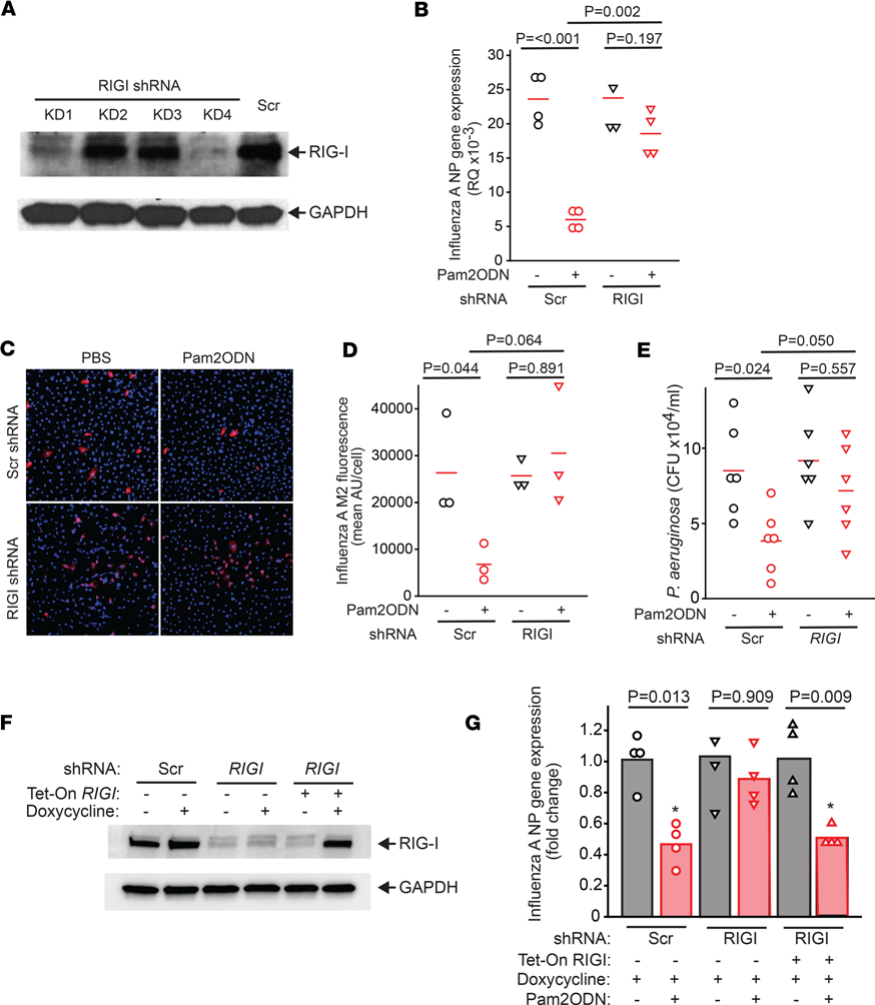
RIG-I is required for Pam2ODN-inducible lung epithelial resistance in vitro. (**A**) Efficacy of RIG-I shRNA knockdown. HBEC3-KT cells were transduced with the indicated shRNA and then immunoblotted for RIG-I. Stable knockdown no. 4 (KD4) was used in subsequent pathogen killing assays. (**B**–**D**) Viral burden of RIG-I shRNA–knockdown HEBC3-KT cells was assessed 24 hours after infection with influenza A virus by (**B**) qPCR of viral nucleocapsid NP gene expression or (**C** and **D**) viral M2 protein immunofluorescence staining. Original magnification, ×10. (**E**) Bacterial burden 6 hours after *P*. *aeruginosa* challenge in RIG-I–knockdown cells was measured by counting CFUs. (**F**) RIG-I shRNA–knockdown HBEC3-KT cells were transduced with a tetracycline-inducible human *RIGI* cDNA construct containing silent mutations in the shRNA targeting sequence. Efficiency of doxycycline-induced RIG-I rescue was determined by immunoblotting. (**G**) Influenza viral burden in doxycycline-treated scrambled or RIG-I shRNA–knockdown or RIG-I–rescued cells with or without Pam2ODN treatment by NP qPCR. *P* values indicate comparison by 1-way ANOVA (Holm-Šidák method). Tet-On *RIGI*, tetracycline-inducible *RIGI* construct; Scr, scrambled shRNA.

To better understand the role of RIG-I in inducible epithelial resistance, we knocked down *RIGI* expression in HBEC3-KT cells (Figure 4A and Supplemental Figure 5) by shRNA and then infected knockdown cells with influenza A virus or *P*. *aeruginosa* in vitro after PBS or Pam2ODN treatment. Whether quantified by viral nucleocapsid (NP) gene expression or immunofluorescence labeling of viral M2 protein (Figure 4, B–D), Pam2ODN induced significant reductions of viral burden in sham-knockdown cells 24 hours after infection, but this effect was lost in RIG-I–knockdown cells. Although RIG-I activation has been previously associated with antiviral responses (23, 24, 28), we did not observe baseline impairment of virus burden in control cells at the timescales observed. Consistent with our in vivo observation, we also observed loss of Pam2ODN-inducible antipseudomonal responses in *RIGI*-knockdown epithelial cells (Figure 4E).

To further substantiate the specificity of the RIG-I requirement for Pam2ODN protection, we developed a doxycycline-inducible *RIGI* rescue strategy that we applied to *RIGI* shRNA–knockdown HBEC3-KT cells (Figure 4F and Supplemental Figure 5). As shown in Figure 4G, while Pam2ODN-induced protection was impaired in *RIGI*-knockdown cells, doxycycline-induced *RIGI* expression restored the protective phenotype to that observed in sham-knockdown cells. This indicates that ODN-dependent RIG-I activation is required for therapeutically inducible antiviral protection.

### ODN induces MAVS-dependent pneumonia-protective signaling.

**Figure 5:**
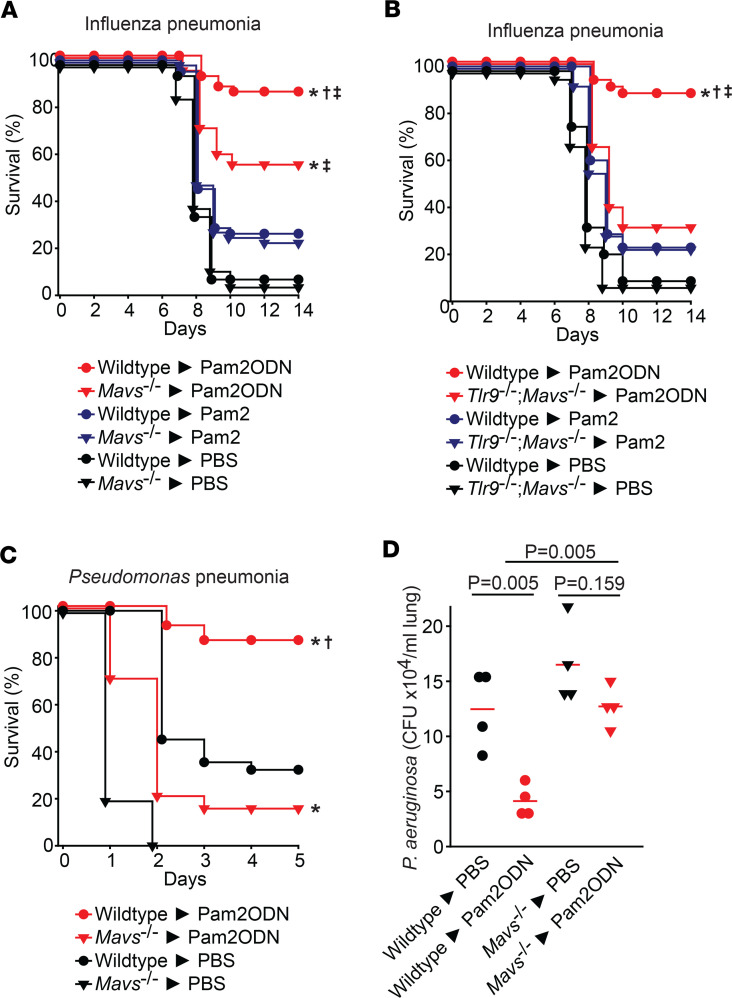
MAVS is required for ODN protection against pneumonia. (**A**) Wild-type or *Mavs^–/–^* mice or (**B**) wild-type or *Tlr9^–/–^*;*Mavs^–/–^* double-knockout mice were challenged with lethal doses of influenza A virus 24 hours after nebulized treatment with PBS, Pam2, or Pam2ODN. (**C**) Wild-type or *Mavs^–/–^* mice were challenged with lethal doses of *P*. *aeruginosa* 24 hours after nebulized treatment with PBS or Pam2ODN. (**D**) Bacterial burden of lungs in **C**, compared by 1-way ANOVA (Holm-Šidák method). *P < 0.01 vs. PBS-treated syngeneic mice, †*P* < 0.01 vs. Pam2ODN-treated knockout mice, ‡*P* < 0.01 vs. Pam2-treated syngeneic mice, all by log-rank test (Mantel-Cox method).

Upon binding its native 5′ triphosphate hairpin RNA ligand, RIG-I activation promotes aggregation of MAVS (23, 24). Immunoblotting demonstrates a similar pattern of MAVS aggregation following treatment of HBEC3-KT cells with 5′ triphosphate hairpin RNA or ODN (Supplemental Figure 6), suggesting that MAVS may also be required for ODN-induced RIG-I signaling. To formally test this, *Mavs^–/–^* and wild-type mice were treated with PBS, Pam2, or Pam2ODN 24 hours prior to influenza A virus. Pam2ODN-treated MAVS-deficient mice displayed a partial protection phenotype (Figure 5A) that paralleled that observed in Pam2ODN-treated *Tlr9^–/–^* or *Rigi^–/–^* mice and supported a role for MAVS in ODN-induced antiviral responses. *Tlr9^–/–^*;*Mavs^–/–^* double-knockout mice phenocopied *Tlr9^–/–^***;***Rigi^–/–^* mice, as they demonstrated no survival advantage of influenza A challenge following Pam2ODN treatment (Figure 5B), indicating a critical role for MAVS signaling in inducible resistance to viral infection. Furthermore, congruent with our surprising observation that RIG-I is required for Pam2ODN-induced antibacterial protection, we found that MAVS is also required for full inducible protection against *P*. *aeruginosa* infection (Figure 5, C and D).

### ODN protection requires both TLR9 and RIG-I pneumonia-protective signaling.

**Figure 6:**
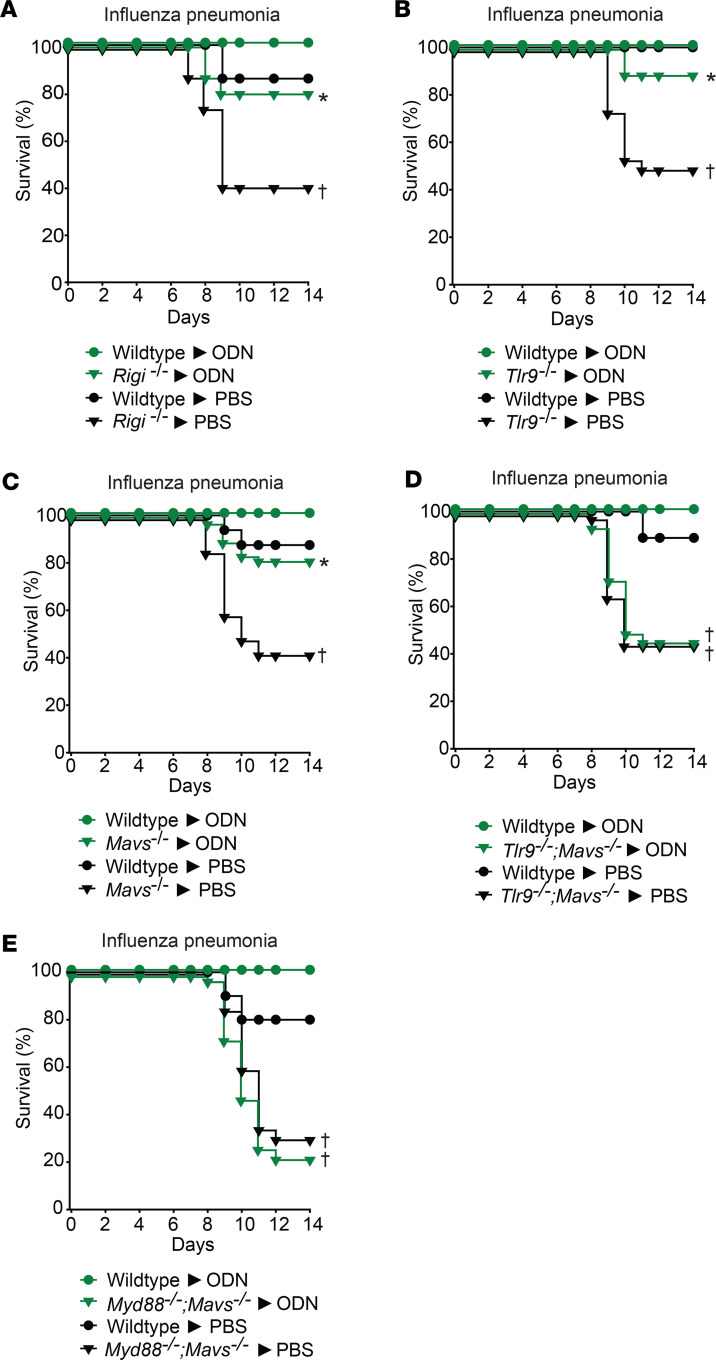
Single-ligand ODN treatment induced protection against viral pneumonia in TLR9, RIG-I, MyD88, and MAVS mutant mice. (**A**) *Rigi^–/–^* or wild-type littermates, (**B**) wild-type mice and *Tlr9^–/–^* mice, (**C**) *Mavs^–/–^* mice, (**D**) *Tlr9^–/–^*;*Mavs^–/–^* mice, or (**E**) *MyD88^–/–^*;*Mavs^–/–^* mice were challenged with inhaled influenza A virus at an inoculum that is generally sublethal in wild-type mice (2 × 10^4^ PFU) 24 hours after nebulized treatment with PBS or single-ligand ODN. Shown is mouse survival after infection. *P < 0.05 vs. PBS-treated syngeneic mice, †*P* < 0.001 vs. PBS-treated wild-type mice by log-rank test (Mantel-Cox method).

Synergy between Pam2 and ODN is a cardinal feature of inducible resistance, and this interaction affords maximal protection against otherwise lethal infections (as above in Figure 1 and refs. 8–16). However, exclusively testing combination treatments makes it difficult to precisely investigate the roles of individual agents. Since we have repeatedly shown that ODN alone confers a modest degree of protection against highly virulent infections (1.6 × 10^5^ PFU), we developed less lethal models of influenza A pneumonia (Supplemental Figure 7) to better isolate the effect of ODN. In wild-type mice, almost all mice survived low-inoculum influenza A challenges (2 × 10^4^ PFU), regardless of whether they received prior treatment with PBS or ODN. In contrast, mice deficient in TLR9, RIG-I, or MAVS demonstrated significant mortality following low-inoculum challenge, revealing an increase in baseline viral susceptibility. However, high-dose, single-ligand ODN treatment improved influenza A survival of *Tlr9^–/–^*, *Rigi^–/–^*, and *Mavs^–/–^* mice (Figure 6, A–C), whereas *Tlr9^–/–^*;*Mavs^–/–^* and *MyD88^–/–^*;*Mavs^–/–^* double-knockout mice displayed both increased baseline susceptibility and total loss of ODN-inducible protection (Figure 6, D and E). Together, these data reinforce the contributions of both TLR9- and RIG-I–dependent virus-protective responses to ODN.

**Figure 7:**
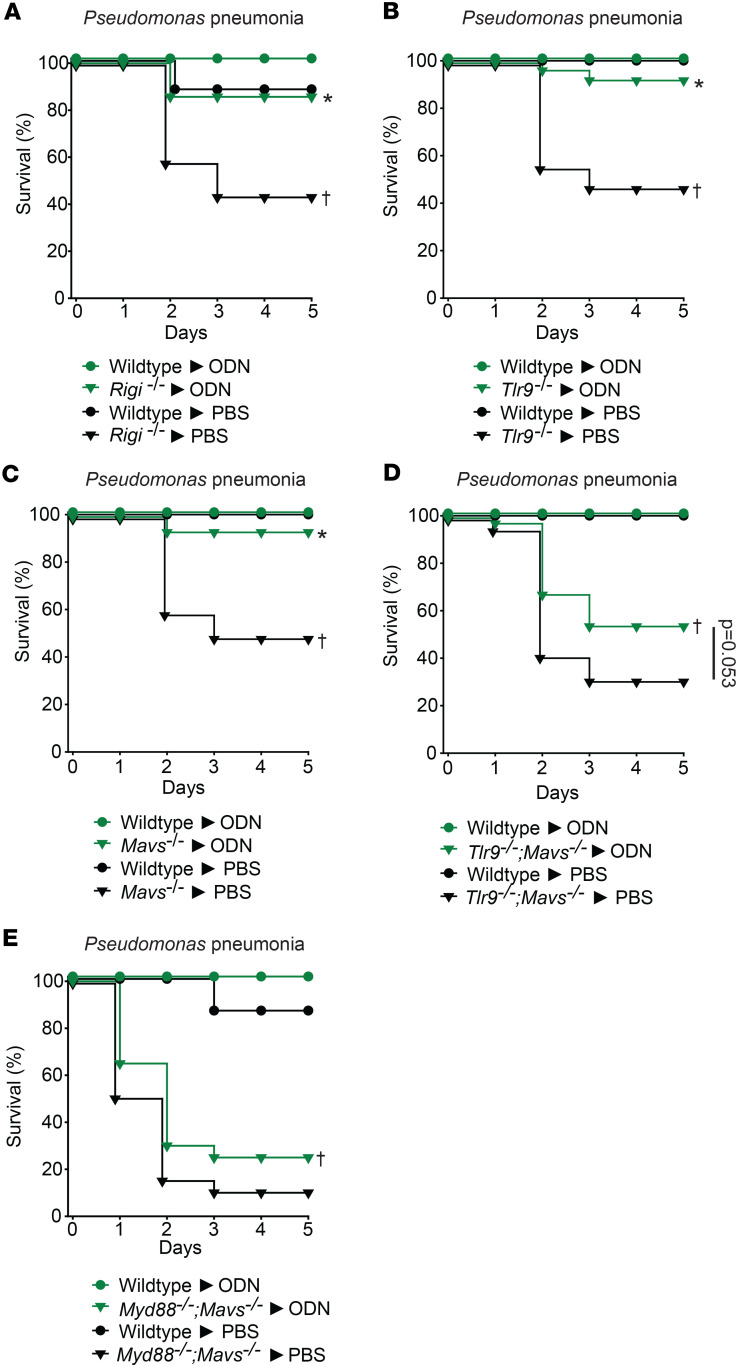
Single-ligand ODN treatment induced protection against bacterial pneumonia in TLR9, RIG-I, MyD88, and MAVS mutant mice. (**A**) *Rigi^–/–^* or wild-type littermates, (**B**) wild-type mice and *Tlr9^–/–^* mice, (**C**) *Mavs^–/–^* mice, (**D**) *Tlr9^–/–^*;*Mavs^–/–^* mice, or (**E**) *MyD88^–/–^*;*Mavs^–/–^* mice were challenged with inhaled *P*. *aeruginosa* at an inoculum that is sublethal in wild-type mice (2.5 × 10^9^ CFU/mL) 24 hours after nebulized treatment with PBS or single-ligand ODN. Shown is mouse survival after infection. *P < 0.05 vs. PBS-treated syngeneic mice, †*P* < 0.001 vs. PBS-treated wild-type mice by log-rank test (Mantel-Cox method).

To further substantiate our observation that RIG-I/MAVS pathway activation is required for Pam2ODN-induced antibacterial protection, we developed a similar model of pseudomonal pneumonia with reduced lethality (2 × 10^10^ CFU/mL vs. 2.5 × 10^9^ CFU/mL) (29). As shown in Figure 7, while single-ligand ODN treatment significantly improved pseudomonal pneumonia survival of *Tlr9^–/–^*, *Rigi^–/–^*, and *Mavs^–/–^* mice, the same ODN treatment did not induce significant protection against pseudomonal pneumonia in *Tlr9^–/–^;Mavs^–/–^ or MyD88^–/–^;Mavs^–/–^* double-knockout mice. Taken together, these results indicate that ODN can protectively stimulate both the TLR9/MyD88 and RIG-I/MAVS pathways, that both pathways are required for maximal ODN protection, and that loss of both completely abrogates the ODN benefit.

### Pam2ODN synergy requires both TLR and RLR signaling activation.

To investigate the role of RIG-I/MAVS pathway activation in ODN-induced protection in vivo, we examined RIG-I–dependent cytokine and interferon induction in mouse lungs upon ODN treatment. Compared with wild-type mice, *Tlr9^–/–^*;*Mavs^–/–^* or *MyD88^–/–^*;*Mavs^–/–^* double-knockout mice displayed loss of ODN-induced cytokine and type I interferon production (Supplemental Figure 8). We have previously reported that ODN-induced mitochondrial ROS generation is essential to the protective antimicrobial effects (22). However, we observed no change of Pam2ODN-induced mitochondrial ROS formation in *RIGI* or *MAVS* shRNA–knockdown HBEC3-KT cells (Supplemental Figure 9), indicating that RIG-I/MAVS activation is not required for ODN-induced voltage-dependent anion channel 1–dependent (VDAC1-dependent) mitochondrial ROS generation that we have previously described (22).

**Figure 8:**
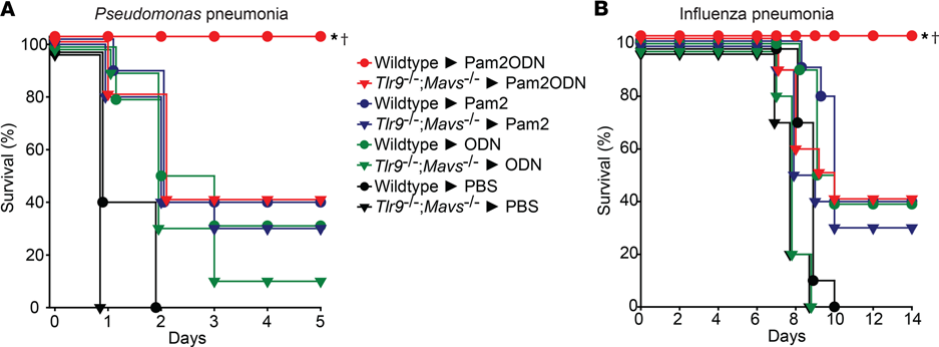
Pam2ODN synergy requires activation of both TLR and RLR signaling pathways. Wild-type or *Tlr9^–/–^*;*Mavs^–/–^* mice were challenged with lethal doses of (**A**) *P*. *aeruginosa* or (**B**) influenza A virus 24 hours after nebulized treatment with PBS, Pam2, ODN, or Pam2ODN. *P < 0.001 vs. PBS-treated syngeneic mice, †*P* < 0.005 vs. Pam2ODN-treated *Tlr9^–/–^*;*Mavs^–/–^* mice by log-rank test (Mantel-Cox method).

Since Pam2 and ODN both are known to activate TLR signaling pathways, but disruption of TLR9 signaling alone is not sufficient to abrogate the synergistic interaction, we hypothesized that Pam2ODN-induced synergy requires activation of the RIG-I/MAVS pathway. To test this hypothesis, wild-type or *Tlr9^–/–^;Mavs^–/–^* mice received a single aerosolized treatment of PBS, Pam2, ODN, or Pam2ODN 24 hours prior to challenge with lethal inocula of *P*. *aeruginosa* or influenza A virus (Figure 8). Similar to our findings in Figure 1, Pam2ODN significantly protected wild-type mice against pneumonia-related death, with each individual ligand affording modest survival advantage. In contrast to the partial protection phenotype observed in *Tlr9^–/–^*, *Rigi^–/–^*, or *Mavs^–/–^* mice, Pam2ODN treatment in the *Tlr9^–/–^;Mavs^–/–^* mice led to no greater protection than Pam2 treatment.

## Discussion

In this work, we found that the RNA sensor RIG-I can also bind DNA-like ODN, activating MAVS-dependent antimicrobial signaling events. RIG-I binding ODN promotes pathogen killing and pneumonia survival both in vitro and in vivo. This study reveals what we believe to be a novel cytosolic PRR for synthetic ODNs that contributes to therapeutically inducible pneumonia-protective antimicrobial responses.

RIG-I is well-established to bind 5′-triphosphorylated short, blunt-ended dsRNA during cytosolic replication of positive- or negative-strand RNA viruses, initiating MAVS-dependent signaling that triggers production of type I interferon and proinflammatory cytokines that are required for innate antiviral responses (23, 24). Here, we introduce an exogenously administered, DNA-like RIG-I binding partner that stimulates unanticipated RIG-I/MAVS-dependent host defense responses against viral and bacterial pneumonia. ODN-induced RIG-I–dependent protection is observed both from cultured human lung epithelial cells and in mice. Thus, here we show that ODN can protectively interact with both its canonical endosomal receptor, TLR9, as well as a cytosolic receptor, RIG-I, in mammals. A prior report from Su and colleagues that identified an interaction between CpG ODN and RIG-I in grass carp suggests broader conservation of this phenomenon (30).

We have previously reported the pneumonia-protective synergy elicited by concomitant treatment with Pam2 and ODN. This therapeutic interaction is curious, because the known receptors for the two ligands (TLR2/6, Pam2; TLR9, ODN) both signal through the same MyD88-dependent pathways, suggesting that their combined effect might be expected to be, at most, additive. The identification of a TLR9- and MyD88-independent ODN receptor provides at least partial explanation for how these two ligands can achieve greater-than-additive effects. This is supported in the current work by the partial loss of protective synergy in the absence of TLR9, RIG-I, or MAVS and total loss of ODN-induced protection when both the TLR9/MyD88 and RIG-I/MAVS pathways are disrupted. These findings demonstrate that Pam2ODN synergy requires activation of intact TLR and RLR signaling pathways. In the current work, only preventative treatments were tested for protection, but prior reports also demonstrate a survival advantage when Pam2ODN is delivered after an infectious challenge (14, 31).

Although both synthetic ODN and native RNA ligands bind RIG-I with similar affinity and both induce MAVS-dependent antiviral events, there appear to be some differences in the nature of the interactions. This is underscored by differences in the effect of ATP or ADP-AlF_4_ exposure on RIG-I binding affinity to ODN and its native RNA ligand, measured by fluorescence polarization anisotropy. It is likely also reflected by the fact that RNA-stimulated RIG-I activation has not been reported to induce antibacterial responses, whereas ODN clearly (and surprisingly) promotes RIG-I–dependent antibacterial protection both in vitro and in vivo. Interactions between RIG-I and bacterial species have been previously described. For example, RIG-I/MAVS-dependent signaling may be stimulated by RNA derived from commensal enteric bacteria to protect against experimentally induced colitis (32). Alternately, transcription of 60–100 base pair poly(dA:dT) oligonucleotide templates to RNA intermediates by RNA polymerase III can activate RIG-I (25, 26). However, the phenomenon here is substantially different in that the RIG-I/MAVS response is directly induced by a DNA-like molecule. This inducible resistance is not due to transcription of ODN sequences to RNA but to the direct binding of 25-base ODN by RIG-I. Further distancing this phenomenon from any dependence on pathogen RNA, we have repeatedly shown Pam2ODN-induced protection to be largely pathogen agnostic (8–16). Moreover, RIG-I activation has been reported in *Mycobacterium tuberculosis* infection models (33–35), further suggesting a role for RIG-I in native antibacterial functions. It is further speculated that our group’s prior reports that bacterial lysates are sufficient to induce epithelial antimicrobial responses may also reflect interactions of bacterial DNA and RIG-I (15, 36, 37).

Since the discovery of RIG-I’s antiviral function, a number of small synthetic RNA ligands have gained attention for potential use in prevention or treatment of viral infections (38). However, exogenously administered RNA molecules always face intrinsic challenges in vivo. First, RNA can be rapidly degraded by ubiquitous RNases. Second, delivery of RNAs across hydrophobic cytosolic membranes presents greater challenges than delivery of DNA-like molecules. In contrast, synthetic ODNs have superior biostability, bioavailability, and biodistribution in animals and humans (39). Furthermore, we have demonstrated that in vitro Pam2ODN administration to human lung epithelial cells and aerosolized treatments to mice (8, 9, 11–14, 16) and humans (ClinicalTrials.gov, NCT04313023, NCT04312997, NCT03794557, NCT02566252, NCT02124278) are well tolerated. The current studies reveal a cytosolic PRR for ODNs that contributes to broad, therapeutically inducible antimicrobial responses. Taken together, these findings not only enhance our understanding of the mechanisms underlying Pam2ODN-induced resistance, but also support RIG-I as a potentially druggable target to broadly protect patients against lethal pneumonias during periods of peak vulnerability.

## Methods

### Sex as a biological variable.

Each experiment was performed with mice of a single sex to reduce intragroup variation. However, every presented experiment was performed with groups comprising both sexes. No sex-dependent variation was found in our experiments.

### Pathogens.

Mouse-adapted influenza A/Hong Kong/8/68 virus (H3N2) was provided by Brian E. Gilbert (Baylor College of Medicine, Houston, Texas, USA). *P*. *aeruginosa* strain PA103 was purchased from ATCC and stored as frozen stock in 20% glycerol in Luria-Bertani medium.

### Cell lines.

Human bronchial epithelial (HBEC3-KT) cells were provided by John Minna (University of Texas Southwestern Medical Center, Dallas, Texas, USA). Murine lung epithelial (MLE-15) cells were provided by Jeffrey Whitsett, Cincinnati Children’s Hospital, Cincinnati, Ohio, USA. HBEC3-KT and MLE-15 cells were authenticated by the UT MD Anderson Characterized Cell Line Core Facility and IDEXX Bioresearch, respectively. HBEC3-KT cells were cultured in keratinocyte serum-free medium supplemented with human epidermal growth factor and bovine pituitary extract (Thermo Fisher Scientific). MLE-15 cells were cultured in DMEM/F2 medium supplemented with 2% of fetal bovine serum and 0.5% of Insulin-Transferrin-Selenium (Thermo Fisher Scientific). Cell cultures were maintained in the presence of 1% of penicillin/streptomycin and glutamine, except during bacterial challenges. All cells were cultured at 37°C with 5% CO_2_. All human cell experiments were performed in accordance with Institutional Review Board of The University of Texas MD Anderson Cancer Center (MDACC).

### Mice.

Wild-type C57BL/6J mice were purchased from The Jackson Laboratory. *MyD88^–/–^* and *Mavs**^–/–^*** mice were purchased from The Jackson Laboratory. *Tlr9**^–/–^*** and *Rigi**^–/–^*** mice were provided by Shizuo Akira (Osaka University, Suita, Osaka, Japan). Each individual experiment was performed with 6- to 8-week-old mice of a single sex in a BSL2 biohazard lab, though all experiments were repeated in mice of both sexes. All mouse experiments were performed in accordance with the MDACC Institutional Animal Care and Use Committee.

### In vivo Pam2ODN nebulization.

Combined 4 μM Pam2 (Invivogen) and 1 μM ODN M362 (Invivogen) in 10 mL 1× PBS was placed in an Aerotech II nebulizer (Biodex) and delivered to unrestrained mice in an exposure chamber via an influx polyethylene tube. With the nebulization of ODN alone, 5 μM ODN M362 was delivered. Nebulization was driven by 10 L/min air supplemented with 5% CO_2_. The exposure chamber connects with an identical efflux polyethylene tube with a low-resistance microbial filter (BB50T, Pall) at its end vented to a biosafety hood (13).

### In vivo influenza infection.

Frozen stock (2.8 × 10^7^ 50% tissue culture infective doses [TCID_50_] ml^−1^, 2 × 10^7^ PFU/mL) of virus was diluted 1:1,250 in 0.05% gelatin in 10 mL 1× PBS and delivered by aerosolization for 45 minutes (1.6 × 10^5^ PFU) to achieve the 90% lethal dose (LD90) to LD100 (100 TCID_50_ per mouse). In the low dose viral infection model, 2 × 10^4^ PFU of virus was nebulized. After infection, animals were weighed daily for at least 14 days and sacrificed if they met euthanasia criteria, which includes signs of respiratory distress or loss of 25% preinfection body weight. At least 8 mice per condition were evaluated for survival analysis. Challenges were performed a minimum of 3 times (11).

### In vivo bacterial infection.

One mL of *P*. *aeruginosa* strain PA103 frozen stock (1 × 10^8^ CFU/mL) was incubated overnight in 100 mL of Tryptic Soy Broth (Millipore) at 37°C with 5% CO_2_ and then expanded in 1 liter of fresh Luria-Bertani media at 37°C to OD 600 of 0.52. Bacterial suspensions were centrifuged, washed, resuspended in 1× PBS, and aerosolized using the same nebulization system described for Pam2ODN treatment. A nebulized inoculum of 10 mL of 2 × 10^10^ CFU/mL was delivered. In the low-dose *P*. *aeruginosa* infection model, 2.5 × 10^9^ CFU/mL of bacteria was nebulized (29). The infected mice were closely monitored for at least 6 days. The relevant euthanasia-triggering criteria consist of any evidence of distressed behaviors, including hypothermia, impaired mobility, respiratory distress, or inability to access food or water. When mice were identified to meet the criteria, they were subjected to euthanasia. At least 8 mice per group were evaluated for survival analysis. Challenges were performed a minimum of 3 times (13).

### Bacterial burden measurement.

HBEC3-KT cells or MLE-15 cells were cultured on 6-well plates in complete media until cell growth reached approximately 80% confluence. Cells were replaced with fresh, antibiotic-free media containing PBS, Pam2, ODN, or Pam2ODN. The final concentrations of Pam2 and ODN in media were 2.4 μM and 0.6 μM, respectively. Four hours after the treatment, 20 μL of *P*. *aeruginosa* PA103 (1 × 10^5^ CFU/mL) was added to each culture well. Four hours after bacteria inoculation, 20 μL of supernatant from each well was aspirated, serially diluted, plated on a Tryptic Soy Broth agar plate and incubated for 16 hours at 37°C. Bacterial CFUs were counted after the incubation. Studies were performed a minimum of 3 times with 4 biological replicates per condition.

### Viral burden measurement.

To measure transcript levels of influenza A virus nucleocapsid protein (*np1*), sample RNA was extracted using a RNeasy extraction kit (Qiagen). 500 ng total RNA was reverse transcribed to cDNA by using an iScript cDNA synthesis kit (Bio-Rad) and submitted to quantitative real time-PCR (qRT-PCR) analysis with SYBR green PCR master mix (Thermo, Fisher). Host 18S rRNA was probed to determine relative expression of viral transcripts.

### Immunoblotting and immunoprecipitation.

HBEC3-KT cells or MLE-15 cells treated with ODN M362 or 3p-hpRNA (Invivogen) were suspended in NP-40 lysis buffer containing Halt protease and phosphatase inhibitor cocktail (Millipore), disrupted by sonication, and extracted at 4°C for 30 minutes. The protein concentration of the lysate was determined using bicinchoninic acid protein assay (Pierce). 50 μg protein in 1× Laemmli buffer was separated by SDS-PAGE and then transferred onto polyvinylidene difluoride (PVDF) membranes (Millipore). The PVDF membranes were blotted with primary antibodies, detected by secondary antibodies with conjugated horseradish peroxidase, and developed using a Pico-sensitive chemiluminescence kit (Pierce). All membranes were stripped and reblotted for GAPDH (14C10, Cell Signaling) as a loading control.

Whole cell lysates were prepared with unlabeled ODN-treated or biotinylated ODN-treated HBEC3-KT cells or MLE-15 cells. To precipitate proteins bound by biotinylated ODN in vivo, streptavidin beads (Pierce) were incubated with whole cell or mitochondria lysates containing 300 μg protein overnight at 4°C under constant gentle rotating. After incubation, streptavidin beads were centrifuged, washed with 1× PBS containing 0.05% Tween-20, resuspended in 50 μL of 2× SDS loading buffer, and boiled for 10 minutes. Elutes from the streptavidin beads were loaded onto SDS PAGE gel (Bio-Rad) and immunoblotted with RIG-I antibody (D-12, Santa Cruz Biotech). Cytosolic lysates made from unlabeled ODN- or FITC-labeled ODN-treated HBEC3-KT cells or MLE-15 cells were subjected to immunoprecipitation with a mouse anti–RIG-I antibody (clone 1C3, Sigma-Aldrich) or IgG isotype control. FITC-labeled ODN was detected by direct FITC fluorescence on the SDS PAGE gel using LI-COR imaging system. Immunoprecipitated RIG-I protein was blotted by a rabbit anti–RIG-I antibody (D14G6, Cell Signaling).

### Purification of hrRIG-I (amino acids 236–925).

Briefly, N-terminally truncated human RIG-I (amino acids 236–925), lacking the CARDs) was expressed with a cleavable N-terminal His-tag in Hi5 insect cells. The protein was purified using a first round of nickel-nitrilotriacetic acid (Ni NTA), followed by tag-cleavage by tobacco etch virus (TEV) protease and a second round of Ni NTA to remove the His tagged TEV. The protein was further purified using a HiTrap SP HP cation exchange chromatography column.

### Electrophoretic mobility shift assay.

Two μL of biotinylated ODN M362 (2.5 μM) was mixed with 2 μL hRIG-I 236–925 protein from 0–32 μM and incubated for 30 minutes on ice. 1 μL of native loading dye (xylene methylene blue, TBE, glycerol 20%) 5x was added to the mix for a total volume of 5 μL. Half of the sample (2.5 μL) was loaded on the 6% acrylamide native gel. The gel was prewarmed at 150 V in 0.5x TBE buffer before sample loading and then run at 90 V for 40 minutes. The gel was stained in SYBR Gold in 0.5x TBE for 10 minutes. SYBR gold is a sensitive fluorescent stain that detects DNA and RNA. The Bio-Rad Gel Doc XR+ System with blue light was used to detect fluorescence of hRIG-I bound to ODNM362.

### Fluorescence polarization anisotropy.

hRIG-I 236 was titrated into 5′ FAM labeled ODN or 12-mer dsRNA by annealing 5′ FAM labeled 12-mer RNA with unlabeled cRNA (5′-FAMCAUGUGGAGCCC-3′ and 5′-pppGGGCUCCACAUG-3, respectively) in 20 mM Hepes, 2 mM EDTA at pH 6.8. Labeled DNA and RNA were adjusted to 2.5 nM in anisotropy buffer (20 mM Hepes pH7.5, 150 mM NaCl, 2 mM TCEP, 4 mM MgCl2, 5% glycerol). 79 μL was placed in a 384-well black Greiner microplate. Protein was titrated by adding 1 μL of increasing protein concentration in a final reaction volume of 80 μL. Various nucleotides, ADP-AlF_4_, ADP, or ATP, were added into the reaction mixture. Fluorescence polarization was measured at 25°C with a microplate reader (CLARIOstar, BMG LABTECH) using an excitation wavelength of 495 nm and emission wavelength of 515 nm. For analysis, the polarization value for the oligonucleotide alone was subtracted from the measurements. Experiments were performed at least 3 times. Binding data were analyzed by GraphPad Prism software and fitted with the following equation,
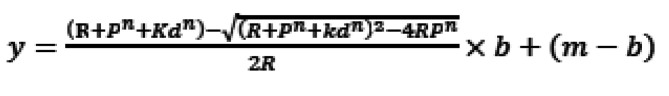


where *y* stands for experimental anisotropy, *b* denotes initial anisotropy, *m* represents the maximum anisotropy, *R* signifies the RNA concentration, *P* indicates the protein concentration, *K*d denotes equilibrium binding constant, and *n* represents the Hill coefficient.*Lentiviral shRNA knockdown and Tet-On rescue*. GIPZ *E*. *coli* clones containing human *RIGI* and *MAVS* lentiviral shRNA vectors were purchased from GE Dharmarcon. The lentiviral shRNA vectors were purified using a QIAGEN plasmid kit. Lentiviruses bearing human *RIGI* and *MAVS* shRNA were produced by cotransfection of the lentiviral shRNA vectors and lentiviral packaging vectors in 293T cells. The shRNA lentiviruses were collected and added into HBEC3-KT cell culture. Lentivirus-infected HBEC3-KT cells were selected by cell sorting based on GFP expression 3 days after lentiviral infection. Efficiency of the shRNA knockdown was determined by immunoblotting with anti-human RIG-I antibody (D-12, Santa Cruz Biotech) and MAVS antibody (E-3, Santa Cruz Biotech).

A human *RIGI* wild-type cDNA (Addgene plasmid 167289) fragment, in which the RIG-I shRNA targeting sequence 5′- *tcagagatagtcaagaaaa*-3′ was site-directed mutated into 5′-*tcgagactcccaggaaaaa*-3′ without change of the encoded protein sequence (Takara Bio), was inserted into a Tet-On pLenti CMVtight hygro vector (Addgene plasmid 26585). After lentiviral infection of the pLenti *RIGI* wild-type cDNA in the correspondent RIG-I shRNA–knockdown HBEC3-KT cells, the stable cell lines were selected by 50 μg/mL hygromycin B (Sigma-Aldrich) for 3 days. The wild-type *RIGI* rescued cells were treated with 0.5 mg/mL of doxycycline (Sigma-Aldrich) to induce RIG-I expression. Efficiency of the RIG-I rescue was determined by immunoblotting with the anti-human RIG-I antibody (D-12, Santa Cruz Biotech).

### Quantitative RT-PCR.

Transcript (mRNA) levels of influenza *np1* gene (primers 5′-CTCATCCTTTATGACAAAGAAG-3′ and 5′-AGATCATCATGTGAGTCAGAC-3′), mouse *Cxcl 10* (primers 5′-CCAAGTGCTGCCGTCATTTTC-3′ and 5′- TCCCTATGGCCCTCATTCTCA-3′), and mouse *5s rRNA* gene (26) were measured by qRT-PCR. Total RNA was isolated from treated cells or mouse lungs using an RNAeasy extraction kit (Qiagen). 500 ng of total RNA was reverse transcribed to cDNA using an iScript cDNA synthesis Kit (Bio-Rad). The cDNA was quantified by real-time PCR analysis using SYBR green PCR master mix (Applied Biosystems, Life Technologies) and specific gene primers. The human *18S* rRNA (primers 5′-GTAACCCGTTGAACCCCATT-3′ and 5′-CCATCCAATCGGTAGTAGCG-3′) or mouse *GAPDH* (primers 5′-CATCACTGCCACCCAGAAGACTG-3′ and 5′-ATGCCAGTGAGCTTCCCGTTCAG-3′) was used as an internal control to determine relative levels of the gene transcripts in treatment groups. qPCR analysis was carried out on a CFX96 Touch Real-Time PCR Detection System with CFX Maestro Software (Bio-Rad).

### ELISA.

Samples of whole lung homogenate were made from mice 24 hours after ODN treatment. Mouse interferon α1 and β ELISA were performed with kits from BioLegend, following the manufacturer’s instructions.

### Indirect immunofluorescence assay.

PBS- or ODN-treated HBEC3-KT cells growing on a chambered coverglass were fixed with 2% paraformaldehyde, permeabilized with 0.1% Triton X-100, and blocked with 2% goat serum in 1× PBS. Cells were incubated with primary antibodies against influenza A virus M2 (14C2, Santa Cruz Biotech) at a dilution of 1:500 for 1 hour and then with Alexa Fluor secondary antibodies (A-21428, Life Technologies) at a dilution of 1:500 for half an hour, and counterstained with DAPI for 15 minutes. Cells were visualized using a DeltaVision deconvolution fluorescence microscope (GE Life Sciences). Fluorescence intensity of microscope images was quantified using ImageJ (NIH).

### Proteomics analysis.

The biotinylated ODN-bound proteins were precipitated with streptavidin beads from whole cell lysates and resolved by PAGE (Bio-Rad). The PAGE gels were stained using Coomassie blue. Coomassie-stained gel pieces were excised, washed, destained and digested in-gel with 200 ng modified trypsin (sequencing grade, Promega) and Rapigest (Waters Corp.) for 18 hours at 37° C. In-solution samples were precipitated with 5:1 v/v of cold acetone at –20° C for 18 hours and then centrifuged, and the acetone was removed prior to treatment with Rapigest (100 °C for 10 min), followed by addition of trypsin. The resulting peptides were extracted and analyzed by high-sensitivity LC-MS/MS on an Orbitrap Fusion mass spectrometer (Thermo Scientific). Proteins were identified by database searching of the fragment spectra against the SwissProt (EBI) protein database using Mascot (v 2.6, Matrix Science) and Proteome Discoverer (v 2.2, Thermo Scientific). Typical search settings included mass tolerances, 10 ppm precursor, 0.8 d fragments; variable modifications, methionine sulfoxide, pyro-glutamate formation; and enzyme, trypsin, up to 2 missed cleavages. Peptides were subject to 1% false discovery rate using reverse-database searching.

### Statistics.

Statistical analyses were performed using SigmaPlot 14.0 (Systat Software) and GraphPad Prism 8. Means of two groups were compared using 2-tailed Student’s *t* test. Means of multiple groups were compared using 1-way ANOVA. Survival comparisons were performed using log-rank testing by the Mantel-Cox method. *P* values of less than 0.05 were considered significant.

### Study approval.

All mouse experiments were performed in accordance with and with approval from the MDACC Institutional Animal Care and Use Committee. All human cell experiments were performed in accordance with and with approval from the MDACC Institutional Review Board.

### Data and materials availability.

All supporting data are available upon request. Values for all data points in graphs are reported in the Supporting Data Values file.

## Author contributions

Conceptualization was provided by YW and SEE. Methodology was provided by YW and SEE. Formal analysis was provided by JPG, ML, YW, and SEE. Investigation was provided by YW, VVK, JPG, and ML. Resources were provided by SC. Funding was acquired by SEE. Supervision was provided by SEE. The original draft of the manuscript was written by YW and SEE. The manuscript was reviewed and edited by YW, MKL, and SEE.

## Supplementary Material

Supplemental data

Unedited blot and gel images

Supporting data values

## Version 1 10/01/2024

In-Press Preview

## Version 2 11/08/2024

Electronic publication
