# Supplementary material for: The RNA receptor RIG-I binding synthetic oligodeoxynucleotide promotes pneumonia survival
Source: JCI Insight. 2024 Nov 8;9(21):e180584. doi: 10.1172/jci.insight.180584 (PMC11601584; doi:10.1172/jci.insight.180584)

Table S1 Mouse group sizes and survival analyses by Prism

**Figure 1A**								
Genotype	Wildtype	Wildtype	Wildtype	Wildtype	*Tlr9*$^{-/-}$	*Tlr9*$^{-/-}$	*Tlr9*$^{-/-}$	*Tlr9*$^{-/-}$
Treatment	PBS	Pam2	ODN	Pam2ODN	PBS	Pam2	ODN	Pam2ODN
Mouse n #	18	18	18	18	18	18	18	17
Comparison of Survival Curves					Log-rank (Mantel-Cox) test			
Multiple curves					P<0.0001			
Wildtype + PBS vs. Wildtype + Pam2ODN					P<0.0001			
Wildtype + Pam2 vs. Wildtype + Pam2ODN					P<0.0001			
Wildtype + ODN vs. Wildtype + Pam2ODN					P<0.0001			
Tlr9$^{-/-}$ + PBS vs. *Tlr9*$^{-/-}$ + Pam2ODN					P<0.0001			
Tlr9$^{-/-}$ + Pam2 vs. *Tlr9*$^{-/-}$ + Pam2ODN					P=0.0044			
Tlr9$^{-/-}$ + ODN vs. *Tlr9*$^{-/-}$ + Pam2ODN					P<0.0001			
Tlr9$^{-/-}$ + Pam2ODN vs. Wildtype + Pam2ODN					P=0.0261			
Bonferroni-corrected P value					P=0.0071			
**Figure 1B**								
Genotype	Wildtype	Wildtype	Wildtype	Wildtype	*Tlr9*$^{-/-}$	*Tlr9*$^{-/-}$	*Tlr9*$^{-/-}$	*Tlr9*$^{-/-}$
Treatment	PBS	Pam2	ODN	Pam2ODN	PBS	Pam2	ODN	Pam2ODN
Mouse n #	30	30	30	30	30	30	30	30
Comparison of Survival Curves					Log-rank (Mantel-Cox) test			
Multiple curves					P<0.0001			
Wildtype + PBS vs. Wildtype + Pam2ODN					P<0.0001			
Wildtype + Pam2 vs. Wildtype + Pam2ODN					P<0.0001			
Wildtype + ODN vs. Wildtype + Pam2ODN					P<0.0001			
Tlr9$^{-/-}$ + PBS vs. *Tlr9*$^{-/-}$ + Pam2ODN					P<0.0001			
Tlr9$^{-/-}$ + Pam2 vs. *Tlr9*$^{-/-}$ + Pam2ODN					P=0.0055			
Tlr9$^{-/-}$ + ODN vs. *Tlr9*$^{-/-}$ + Pam2ODN					P<0.0001			
Tlr9$^{-/-}$ + Pam2ODN vs. Wildtype + Pam2ODN					P=0.0163			
Bonferroni-corrected P value					P=0.0071			
**Figure 3A**								
Genotype	Wildtype	Wildtype	Wildtype	*Rigi*$^{-/-}$	*Rigi*$^{-/-}$	*Rigi*$^{-/-}$		
Treatment	PBS	Pam2	Pam2ODN	PBS	Pam2	Pam2ODN		
Mouse n #	28	35	38	28	35	38		
Comparison of Survival Curves					Log-rank (Mantel-Cox) test			
Multiple curves					P<0.0001			
Wildtype + PBS vs. Wildtype + Pam2ODN					P<0.0001			
Wildtype + Pam2 vs. Wildtype + Pam2ODN					P<0.0001			
Rigi$^{-/-}$ + PBS vs. *Rigi*$^{-/-}$ + Pam2ODN					P<0.0001			
Rigi$^{-/-}$ + Pam2 vs. *Rigi*$^{-/-}$ + Pam2ODN					P=0.0012			
Rigi$^{-/-}$ + Pam2ODN vs. Wildtype + Pam2ODN					P<0.0001			

Bonferroni-corrected P value					P=0.01			
**Figure 3B**								
Genotype	Wildtype	Wildtype	Wildtype	*Tlr9*$^{-/-}$; *Rigi*$^{-/-}$	*Tlr9*$^{-/-}$; *Rigi* $^{-/-}$	*Tlr9*$^{-/-}$; *Rigi* $^{-/-}$		
Treatment	PBS	Pam2	Pam2ODN	PBS	Pam2	Pam2ODN		
Mouse n #	25	25	25	25	25	27		
Comparison of Survival Curves					Log-rank (Mantel-Cox) test			
Multiple curves					P<0.0001			
Wildtype + PBS vs. Wildtype + Pam2ODN					P<0.0001			
Wildtype + Pam2 vs. Wildtype + Pam2ODN					P<0.0001			
Tlr9$^{-/-}$ *Rigi*$^{-/-}$ + PBS vs. *Tlr9*$^{-/-}$ *Rigi*$^{-/-}$ + Pam2ODN					P<0.0001			
Tlr9$^{-/-}$ *Rigi*$^{-/-}$ + Pam2 vs. *Tlr9*$^{-/-}$ *Rigi*$^{-/-}$ + Pam2ODN					P=0.0656			
Tlr9$^{-/-}$ *Rigi*$^{-/-}$ + Pam2ODN vs. Wildtype + Pam2ODN					P<0.0001			
Bonferroni-corrected P value					P=0.01			
**Figure 3C**								
Genotype	Wildtype	Wildtype	*Rigi*$^{-/-}$	*Rigi* $^{-/-}$				
Treatment	PBS	Pam2ODN	PBS	Pam2ODN				
Mouse n #	38	39	40	46				
Comparison of Survival Curves					Log-rank (Mantel-Cox) test			
Multiple curves					P<0.0001			
Wildtype + PBS vs. Wildtype + Pam2ODN					P<0.0001			
Rigi$^{-/-}$ + PBS vs. *Rigi*$^{-/-}$ + Pam2ODN					P<0.0001			
Rigi$^{-/-}$ + Pam2ODN vs. Wildtype + Pam2ODN					P<0.0001			
Bonferroni-corrected P value					P=0.0167			
**Figure 5A**								
Genotype	Wildtype	Wildtype	Wildtype	*Mavs*$^{-/-}$	*Mavs* $^{-/-}$	*Mavs* $^{-/-}$		
Treatment	PBS	Pam2	Pam2ODN	PBS	Pam2	Pam2ODN		
Mouse n #	30	42	45	30	45	45		
Comparison of Survival Curves					Log-rank (Mantel-Cox) test			
Multiple curves					P<0.0001			
Wildtype + PBS vs. Wildtype + Pam2ODN					P<0.0001			
Wildtype + Pam2 vs. Wildtype + Pam2ODN					P<0.0001			
Mavs$^{-/-}$ + PBS vs. *Mavs*$^{-/-}$ + Pam2ODN					P<0.0001			
Mavs$^{-/-}$ + Pam2 vs. *Mavs*$^{-/-}$ + Pam2ODN					P=0.0018			
Mavs$^{-/-}$ + Pam2ODN vs. Wildtype + Pam2ODN					P=0.0009			
Bonferroni-corrected P value					P=0.01			
**Figure 5B**								
Genotype	Wildtype	Wildtype	Wildtype	*Tlr9*$^{-/-}$;*Mavs* $^{-/-}$	*Tlr9*$^{-/-}$;*Mavs* $^{-/-}$	*Tlr9*$^{-/-}$;*Mavs* $^{-/-}$		

Treatment	PBS	Pam2	Pam2ODN	PBS	Pam2	Pam2ODN		
Mouse n #	35	35	35	35	35	35		
Comparison of Survival Curves					Log-rank (Mantel-Cox) test			
Multiple curves					P<0.0001			
Wildtype + PBS vs. Wildtype + Pam2ODN					P<0.0001			
Wildtype + Pam2 vs. Wildtype + Pam2ODN					P<0.0001			
Tlr9$^{-/-}$ *Mavs*$^{-/-}$ + PBS vs. *Tlr9*$^{-/-}$ *Mavs*$^{-/-}$ + Pam2ODN					P<0.0001			
Tlr9$^{-/-}$ *Mavs*$^{-/-}$ + Pam2 vs. *Tlr9*$^{-/-}$ *Mavs*$^{-/-}$ + Pam2ODN					P=0.2476			
Tlr9$^{-/-}$ *Mavs*$^{-/-}$ + Pam2ODN vs. Wildtype + Pam2ODN					P<0.0001			
Bonferroni-corrected P value					P=0.01			
**Figure 5C**								
Genotype	Wildtype	Wildtype	*Mavs*$^{-/-}$	*Mavs*$^{-/-}$				
Treatment	PBS	Pam2ODN	PBS	Pam2ODN				
Mouse n #	31	32	37	38				
Comparison of Survival Curves					Log-rank (Mantel-Cox) test			
Multiple curves					P<0.0001			
Wildtype + PBS vs. Wildtype + Pam2ODN					P<0.0001			
Mavs$^{-/-}$ + PBS vs. *Mavs*$^{-/-}$ + Pam2ODN					P<0.0001			
Mavs$^{-/-}$ + Pam2ODN vs. Wildtype + Pam2ODN					P<0.0001			
Bonferroni-corrected P value					P=0.0167			
**Figure 6A**								
Genotype	Wildtype	Wildtype	*Tlr9*$^{-/-}$	*Tlr9*$^{-/-}$				
Treatment	PBS	ODN	PBS	ODN				
Mouse n #	8	8	25	25				
Comparison of Survival Curves					Log-rank (Mantel-Cox) test			
Multiple curves					P=0.0003			
Tlr9$^{-/-}$ + PBS vs. *Tlr9*$^{-/-}$ + ODN					P<0.0001			
Tlr9$^{-/-}$ + PBS vs. Wildtype + PBS					P=0.0142			
Bonferroni-corrected P value					P=0.0167			
**Figure 6B**								
Genotype	Wildtype	Wildtype	*Rig-i*$^{-/-}$	*Rig-i*$^{-/-}$				
Treatment	PBS	ODN	PBS	ODN				
Mouse n #	15	15	15	15				
Comparison of Survival Curves					Log-rank (Mantel-Cox) test			
Multiple curves					P=0.0004			
Rig-i$^{-/-}$ + PBS vs. *Rig-i*$^{-/-}$ + ODN					P=0.0330			
Rig-i$^{-/-}$ + PBS vs. Wildtype + PBS					P=0.0063			
Bonferroni-corrected P value					P=0.025			
**Figure 6C**								

Genotype	Wildtype	Wildtype	*Mavs*$^{-/-}$	*Mavs*$^{-/-}$				
Treatment	PBS	ODN	PBS	ODN				
Mouse n #	16	16	49	51				
Comparison of Survival Curves					Log-rank (Mantel-Cox) test			
Multiple curves					P<0.0001			
Mavs$^{-/-}$ + PBS vs. *Mavs*$^{-/-}$ + ODN					P<0.0001			
Mavs$^{-/-}$ + PBS vs. Wildtype + PBS					P=0.0094			
Bonferroni-corrected P value					P=0.025			
**Figure 6D**								
Genotype	Wildtype	Wildtype	*Tlr9*$^{-/-}$;*Mavs*$^{-/-}$	*Tlr9*$^{-/-}$;*Mavs*$^{-/-}$				
Treatment	PBS	ODN	PBS	ODN				
Mouse n #	9	9	27	27				
Comparison of Survival Curves					Log-rank (Mantel-Cox) test			
Multiple curves					P=0.0053			
Tlr9$^{-/-}$ *Mavs*$^{-/-}$ + PBS vs. *Tlr9*$^{-/-}$ *Mavs*$^{-/-}$ + ODN					P=0.94			
Tlr9$^{-/-}$ *Mavs*$^{-/-}$ + PBS vs. Wildtype + PBS					P=0.0234			
Bonferroni-corrected P value					P=0.025			
**Figure 6E**								
Genotype	Wildtype	Wildtype	*MyD88*$^{-/-}$;*Mavs*$^{-/-}$	*MyD88*$^{-/-}$;*Mavs*$^{-/-}$				
Treatment	PBS	ODN	PBS	ODN				
Mouse n #	10	10	24	24				
Comparison of Survival Curves					Log-rank (Mantel-Cox) test			
Multiple curves					P<0.0001			
MyD88$^{-/-}$ *Mavs*$^{-/-}$ + PBS vs. *MyD88*$^{-/-}$ *Mavs*$^{-/-}$ + ODN					P=0.3617			
MyD88$^{-/-}$ *Mavs*$^{-/-}$ + PBS vs. Wildtype + PBS					P=0.0190			
Bonferroni-corrected P value					P=0.025			
**Figure 7A**								
Genotype	Wildtype	Wildtype	*Tlr9*$^{-/-}$	*Tlr9*$^{-/-}$				
Treatment	PBS	ODN	PBS	ODN				
Mouse n #	10	10	24	24				
Comparison of Survival Curves					Log-rank (Mantel-Cox) test			
Multiple curves					P<0.0001			
Tlr9$^{-/-}$ + PBS vs. *Tlr9*$^{-/-}$ + ODN					P=0.0006			
Tlr9$^{-/-}$ + PBS vs. Wildtype + PBS					P=0.0042			
Bonferroni-corrected P value					P=0.025			
**Figure 7B**								
Genotype	Wildtype	Wildtype	*Rig-i*$^{-/-}$	*Rig-i*$^{-/-}$				

Treatment	PBS	ODN	PBS	ODN				
Mouse n #	18	18	21	21				
Comparison of Survival Curves					Log-rank (Mantel-Cox) test			
Multiple curves					P<0.0001			
Rig-i $^{-/-}$ + PBS vs. *Rig-i* $^{-/-}$ + ODN					P=0.0046			
Rig-i $^{-/-}$ + PBS vs. Wildtype + PBS					P=0.0035			
Bonferroni-corrected P value					P=0.025			
**Figure 7C**								
Genotype	Wildtype	Wildtype	*Mavs* $^{-/-}$	*Mavs* $^{-/-}$				
Treatment	PBS	ODN	PBS	ODN				
Mouse n #	20	20	40	40				
Comparison of Survival Curves					Log-rank (Mantel-Cox) test			
Multiple curves					P<0.0001			
Mavs $^{-/-}$ + PBS vs. *Mavs* $^{-/-}$ + ODN					P<0.0001			
Mavs $^{-/-}$ + PBS vs. Wildtype + PBS					P<0.0001			
Bonferroni-corrected P value					P=0.025			
**Figure 7D**								
Genotype	Wildtype	Wildtype	*Tlr9* $^{-/-}$;*Mavs* $^{-/-}$	*Tlr9* $^{-/-}$;*Mavs* $^{-/-}$				
Treatment	PBS	ODN	PBS	ODN				
Mouse n #	10	10	30	30				
Comparison of Survival Curves					Log-rank (Mantel-Cox) test			
Multiple curves					P<0.0001			
Tlr9 $^{-/-}$ *Mavs* $^{-/-}$ + PBS vs. *Tlr9* $^{-/-}$ *Mavs* $^{-/-}$ + ODN					P=0.0526			
Tlr9 $^{-/-}$ *Mavs* $^{-/-}$ + PBS vs. Wildtype + PBS					P=0.0003			
Bonferroni-corrected P value					P=0.025			
**Figure 7E**								
Genotype	Wildtype	Wildtype	*MyD88* $^{-/-}$;*Mavs* $^{-/-}$	*MyD88* $^{-/-}$;*Mavs* $^{-/-}$				
Treatment	PBS	ODN	PBS	ODN				
Mouse n #	16	16	20	20				
Comparison of Survival Curves					Log-rank (Mantel-Cox) test			
Multiple curves					P<0.0001			
MyD88 $^{-/-}$ *Mavs* $^{-/-}$ + PBS vs. *MyD88* $^{-/-}$ *Mavs* $^{-/-}$ + ODN					P=0.1855			
MyD88 $^{-/-}$ *Mavs* $^{-/-}$ + PBS vs. Wildtype + PBS					P<0.0001			
Bonferroni-corrected P value					P=0.025			
**Figure 8A**								
Genotype	Wildtype	Wildtype	Wildtype	Wildtype	*Tlr9* $^{-/-}$;*Mavs* $^{-/-}$	*Tlr9* $^{-/-}$;*Mavs* $^{-/-}$	*Tlr9* $^{-/-}$;*Mavs* $^{-/-}$	*Tlr9* $^{-/-}$;*Mavs* $^{-/-}$

Treatment	PBS	Pam2	ODN	Pam2ODN	PBS	Pam2	ODN	Pam2ODN
Mouse n #	10	10	10	10	10	10	10	10
Comparison of Survival Curves					Log-rank (Mantel-Cox) test			
Multiple curves					P<0.0001			
Wildtype + PBS vs. Wildtype + Pam2ODN					P<0.0001			
Wildtype + Pam2 vs. Wildtype + Pam2ODN					P=0.0046			
Wildtype + ODN vs. Wildtype + Pam2ODN					P=0.0013			
Tlr9$^{-/-}$ *Mavs* $^{-/-}$ + PBS vs. *Tlr9*$^{-/-}$ *Mavs* $^{-/-}$ + Pam2ODN					P=0.0004			
Tlr9$^{-/-}$ *Mavs* $^{-/-}$ + Pam2 vs. *Tlr9*$^{-/-}$ *Mavs* $^{-/-}$ + Pam2ODN					P=0.7336			
Tlr9$^{-/-}$ *Mavs* $^{-/-}$ + ODN vs. *Tlr9*$^{-/-}$ *Mavs* $^{-/-}$ + Pam2ODN					P=0.3598			
Tlr9$^{-/-}$ *Mavs* $^{-/-}$ + Pam2ODN vs. Wildtype + Pam2ODN					P=0.0040			
Bonferroni-corrected P value					P=0.0071			
**Figure 8B**								
Genotype	Wildtype	Wildtype	Wildtype	Wildtype	*Tlr9*$^{-/-}$ *;Mavs* $^{-/-}$	*Tlr9*$^{-/-}$ *;Mavs* $^{-/-}$	*Tlr9*$^{-/-}$ *;Mavs* $^{-/-}$	*Tlr9*$^{-/-}$ *;Mavs* $^{-/-}$
Treatment	PBS	Pam2	ODN	Pam2ODN	PBS	Pam2	ODN	Pam2ODN
Mouse n #	10	10	10	10	10	10	10	10
Comparison of Survival Curves					Log-rank (Mantel-Cox) test			
Multiple curves					P<0.0001			
Wildtype + PBS vs. Wildtype + Pam2ODN					P<0.0001			
Wildtype + Pam2 vs. Wildtype + Pam2ODN					P=0.0046			
Wildtype + ODN vs. Wildtype + Pam2ODN					P=0.0046			
Tlr9$^{-/-}$ *Mavs* $^{-/-}$ + PBS vs. *Tlr9*$^{-/-}$ *Mavs* $^{-/-}$ + Pam2ODN					P=0.0133			
Tlr9$^{-/-}$ *Mavs* $^{-/-}$ + Pam2 vs. *Tlr9*$^{-/-}$ *Mavs* $^{-/-}$ + Pam2ODN					P=0.6560			
Tlr9$^{-/-}$ *Mavs* $^{-/-}$ + ODN vs. *Tlr9*$^{-/-}$ *Mavs* $^{-/-}$ + Pam2ODN					P=0.0176			
Tlr9$^{-/-}$ *Mavs* $^{-/-}$ + Pam2ODN vs. Wildtype + Pam2ODN					P=0.0040			
Bonferroni-corrected P value					P=0.0071			

**Figure S1A**				
Genotype	Wildtype	Wildtype	*Cgas*$^{-/-}$	*Cgas* $^{-/-}$
Treatment	PBS	Pam2ODN	PBS	Pam2ODN
Mouse n #	10	10	8	9
Comparison of Survival Curves			Log-rank (Mantel-Cox) test	
Multiple curves			P<0.0001	
Wildtype + PBS vs. Wildtype + Pam2ODN			P=0.0046	
Cgas$^{-/-}$ + PBS vs. *Cgas*$^{-/-}$ + Pam2ODN			P=0.0007	
Cgas$^{-/-}$ + Pam2ODN vs. Wildtype + Pam2ODN			P=0.4355	
Bonferroni-corrected P value			P=0.0167	
**Figure S1B**				
Genotype	Wildtype	Wildtype	*Cgas* $^{-/-}$	*Cgas* $^{-/-}$

Treatment	PBS	Pam2ODN	PBS	Pam2ODN
Mouse n #	9	10	8	9
Comparison of Survival Curves			Log-rank (Mantel-Cox) test	
Multiple curves			P=0.0352	
Wildtype + PBS vs. Wildtype + Pam2ODN			P=0.0413	
Cgas$^{-/-}$ + PBS vs. *Cgas*$^{-/-}$ + Pam2ODN			P=0.0236	
Cgas$^{-/-}$ + Pam2ODN vs. Wildtype + Pam2ODN			P=0.6056	
Bonferroni-corrected P value			P=0.0167	
**Figure S1C**				
Genotype	Wildtype	Wildtype	*Sting*$^{gt/gt}$	*Sting*$^{gt/gt}$
Treatment	PBS	Pam2ODN	PBS	Pam2ODN
Mouse n #	11	12	12	12
Comparison of Survival Curves			Log-rank (Mantel-Cox) test	
Multiple curves			P=0.0009	
Wildtype + PBS vs. Wildtype + Pam2ODN			P=0.0094	
Sting$^{gt/gt}$ + PBS vs. *Sting*$^{gt/gt}$ + Pam2ODN			P=0.0191	
Sting$^{gt/gt}$ + Pam2ODN vs. Wildtype + Pam2ODN			P=0.0701	
Bonferroni-corrected P value			P=0.0167	
**Figure S1D**				
Genotype	Wildtype	Wildtype	*Sting*$^{gt/gt}$	*Sting*$^{gt/gt}$
Treatment	PBS	Pam2ODN	PBS	Pam2ODN
Mouse n #	8	10	9	10
Comparison of Survival Curves			Log-rank (Mantel-Cox) test	
Multiple curves			P<0.0001	
Wildtype + PBS vs. Wildtype + Pam2ODN			P<0.0001	
Sting$^{gt/gt}$ + PBS vs. *Sting*$^{gt/gt}$ + Pam2ODN			P<0.0001	
Sting$^{gt/gt}$ + Pam2ODN vs. Wildtype + Pam2ODN			P=0.9999	
Bonferroni-corrected P value			P=0.0167	
**Figure S1E**				
Genotype	Wildtype	Wildtype	*Ifi204*$^{+/-}$	*Ifi204*$^{+/-}$
Treatment	PBS	Pam2ODN	PBS	Pam2ODN
Mouse n #	10	10	10	10
Comparison of Survival Curves			Log-rank (Mantel-Cox) test	
Multiple curves			P=0.0007	
Wildtype + PBS vs. Wildtype + Pam2ODN			P=0.0040	
Ifi204$^{+/-}$ + PBS vs. *Ifi204*$^{+/-}$ + Pam2ODN			P=0.0141	
Ifi204$^{+/-}$ + Pam2ODN vs. Wildtype + Pam2ODN			P=0.1246	
Bonferroni-corrected P value			P=0.0167	
**Figure S7A**				
Genotype	Wildtype			
Mouse n #	10			
Comparison of Survival Curves			Log-rank (Mantel-Cox) test	

Multiple curves			P<0.0001	
1 x10^5 PFU vs. 0 PFU			P<0.0001	
5 x10^4 PFU vs. 0 PFU			P=0.0117	
2.5 x10^4 PFU vs. 0 PFU			P=0.1462	
Bonferroni-corrected P value			P=0.0167	
**Figure S7B**				
Genotype	*Tlr9*$^{-/-}$			
Mouse n #	5			
Comparison of Survival Curves			Log-rank (Mantel-Cox) test	
Multiple curves			P<0.0001	
1 x10^5 PFU vs. 0 PFU			P=0.0035	
5 x10^4 PFU vs. 0 PFU			P=0.0035	
2.5 x10^4 PFU vs. 0 PFU			P=0.0126	
Bonferroni-corrected P value			P=0.0167	
**Figure S7C**				
Genotype	*Mavs*$^{-/-}$			
Mouse n #	5			
Comparison of Survival Curves			Log-rank (Mantel-Cox) test	
Multiple curves			P<0.0001	
1 x10^5 PFU vs. 0 PFU			P=0.0035	
5 x10^4 PFU vs. 0 PFU			P=0.0031	
2.5 x10^4 PFU vs. 0 PFU			P=0.0026	
Bonferroni-corrected P value			P=0.0167	

Table S2 Protein candidates precipitated from biotinylated ODN M362 treated cells.

Accession	Score	Mass	Num. of matches	Num. of significant matches	Num. of sequences	Sequence coverage	emPAI	Description
MYH10_HUMAN	466	228858	26	26	18	0.07	0.45	Myosin-10 OS=Homo sapiens GN=MYH10 PE=1 SV=3
TBB6_HUMAN	66	49825	3	3	2	0.06	0.19	Tubulin beta-6 chain OS=Homo sapiens GN=TUBB6 PE=1 SV=1
PYC_HUMAN	54	129551	1	1	1	0.01	0.03	Pyruvate carboxylase, mitochondrial OS=Homo sapiens GN=PC PE=1 SV=2
COPA_HUMAN	44	138258	3	3	3	0.02	0.1	Coatomer subunit alpha OS=Homo sapiens GN=COPA PE=1 SV=2
FAS_HUMAN	42	273254	1	1	1	0	0.02	Fatty acid synthase OS=Homo sapiens GN=FASN PE=1 SV=3
MYL6_HUMAN	42	16919	2	2	2	0.14	0.67	Myosin light polypeptide 6 OS=Homo sapiens GN=MYL6 PE=1 SV=2
ADT1_HUMAN	40	33043	1	1	1	0.03	0.14	ADP/ATP translocase 1 OS=Homo sapiens GN=SLC25A4 PE=1 SV=4
RS16_HUMAN	33	16435	2	2	2	0.12	0.69	40S ribosomal protein S16 OS=Homo sapiens GN=RPS16 PE=1 SV=2
DDX58_HUMAN	31	106531	1	1	1	0.01	0.04	Probable ATP-dependent RNA helicase DDX58 OS=Homo sapiens GN=DDX58 PE=1 SV=2
CENPB_HUMAN	30	65132	2	2	2	0.03	0.15	Major centromere autoantigen B OS=Homo sapiens GN=CENPB PE=1 SV=2
CDKL1_HUMAN	29	41645	1	1	1	0.02	0.11	Cyclin-dependent kinase-like 1 OS=Homo sapiens GN=CDKL1 PE=1 SV=5
R51A2_HUMAN	27	133822	1	1	1	0.01	0.03	RAD51-associated protein 2 OS=Homo sapiens GN=RAD51AP2 PE=1 SV=1
3BHS7_HUMAN	26	40990	1	1	1	0.02	0.11	3 beta-hydroxysteroid dehydrogenase type 7 OS=Homo sapiens GN=HSD3B7 PE=1 SV=2
GRP75_HUMAN	26	73635	1	1	1	0.01	0.06	Stress-70 protein, mitochondrial OS=Homo sapiens GN=HSPA9 PE=1 SV=2
SPTN2_HUMAN	26	271157	1	1	1	0	0.02	Spectrin beta chain, non-erythrocytic 2 OS=Homo sapiens GN=SPTBN2 PE=1 SV=3
FLII_HUMAN	25	144659	1	1	1	0.01	0.03	Protein flightless-1 homolog OS=Homo sapiens GN=FLII PE=1 SV=2
OR1E3_HUMAN	25	38492	1	1	1	0.02	0.12	Olfactory receptor 1E3 OS=Homo sapiens GN=OR1E3 PE=3 SV=2

Supplement Figure Legends

Figure S1. Pam2-ODN treatment protects against infections in *Cgas* and *Sting* knockout mice and *Ifi204* heterozygous mice. Survival of wild type or *Cgas*$^{-/-}$ mice challenged with (**A**) influenza A virus or (**B**) *P. aeruginosa* 24 h after nebulized treatment with PBS or Pam2ODN. Survival of wild type or *Sting*$^{gt/gt}$ mice challenged with (**C**) influenza A virus or (**D**) *P. aeruginosa* one day after nebulized treatment with PBS or Pam2ODN. Survival of wild type or *Ifi204*$^{+/-}$ mice challenged with (**E**) influenza A virus one day after nebulized treatment with PBS or Pam2ODN. * p <0.01 vs. PBS-treated syngeneic mice treated by log rank analysis.

Figure S2. Identification of candidate ODN receptors. (**A**) Streptavidin precipitation from lysates of HBEC3-kt cells treated as indicated were resolved by polyacrylamide gel electrophoresis and Coomassie stained. Boxes indicate bands excised for LC-MS analysis. (**B**) Euler diagram of LC-MS-identified proteins binding to ODN, biotinylated-ODN or both in **A**.

Figure S3. Uncut blots from Figure 2. (A) Uncut blot from Panel 4A. (**B**) Uncut blot from Panel 2B. (**C**) Uncut blots from Panel 2C, LI-COR fluorescent imaging for FITC-labeled ODN (*left*) and immunoblot for RIG-I (*right*). (**D**) Uncut blots from Panel 2D, LI-COR fluorescent imaging for FITC-labeled ODN (*left*) and immunoblot for RIG-I (*right*).

Figure S4. RIG-I interaction with ODN is independent of RNA polymerase III. (A) MLE-15 cells were pre-treated with PBS or RNA polymerase III inhibitor ML-60218 for 8 h, then 5s rRNA transcription was examined by qPCR. (**B**) MLE-15 cells were treated with PBS or ML-60218 for 8 h, then exposed to unlabeled ODN or 3' biotinylated-ODN for 2 h prior to streptavidin pulldown of RIG-I. (**C**) MLE-15 cells were exposed to unlabeled ODN or 5' or 3' biotinylated-ODN for 2 h prior to streptavidin pulldown of RIG-I. * p<0.0001 vs PBS-treated by two-way Student's t test.

Figure S5. Uncut blots from Figure 4.

Figure S6. MAVS aggregation upon RIG-I activation by RNA ligand or ODN. HBEC3-KT cells were treated with a known RIG-I ligand 3p-hpRNA or ODN M362 for the indicated times, then examined by MAVS immunoblotting. The smears above the predicted MAVS band indicate MAVS aggregates. GAPDH was a loading control. 3p-hpRNA, 5' triphosphate hairpin RNA.

Figure S7. Influenza A inoculum-dependent survival of wildtype, *Mavs* or *Tlr9* knockout mice. Wildtype mice (**A**), *Tlr9*$^{-/-}$ mice (**B**) or *Mavs*$^{-/-}$ mice (**C**) were challenged with inhaled influenza A virus at the indicated inocula. Shown is survival after infection.

Figure S8. Single ligand ODN treatment-induced *Cxcl10* expression and type I interferon production in mouse lungs. *Cxcl10* gene expression in lung homogenates from wildtype or *Tlr9*$^{-/-}$;*Mavs*$^{-/-}$ mice (**A**) or *MyD88*$^{-/-}$;*Mavs*$^{-/-}$ mice (**B**) 4 h after nebulized PBS or ODN treatment, measured by qPCR. Interferon α1 (**C**) and β (**D**) in lung homogenates from wildtype or *Tlr9*$^{-/-}$;*Mavs*$^{-/-}$ mice 24 h after nebulized PBS or ODN treatment, measured by ELISA.* p<0.02 vs PBS-treated wildtype by one-way ANOVA (Holm-Sidak method).

Figure S9. RIG-I/ MAVS is not involved in mitochondrial ROS pathway in lung epithelial cells. (A) MitoSOX fluorescence in HBEC3-KT RIG-I knockdown cells or scrambled shRNA control cells, shown 100 minutes after treatment with PBS (sham) or Pam2ODN. (**B**) Efficacy of MAVS shRNA knock down in HBEC3-KT cells was determined by immunoblotting. (**C**) MitoSOX fluorescence in HBEC3-KT MAVS knockdown cells or scambled shRNA control cells, shown

100 minutes after treatment with PBS or Pam2ODN. P values reflect intergroup analyses by one-way ANOVA (Holm-Sidak method).

Wang, Supplemental Figure S1

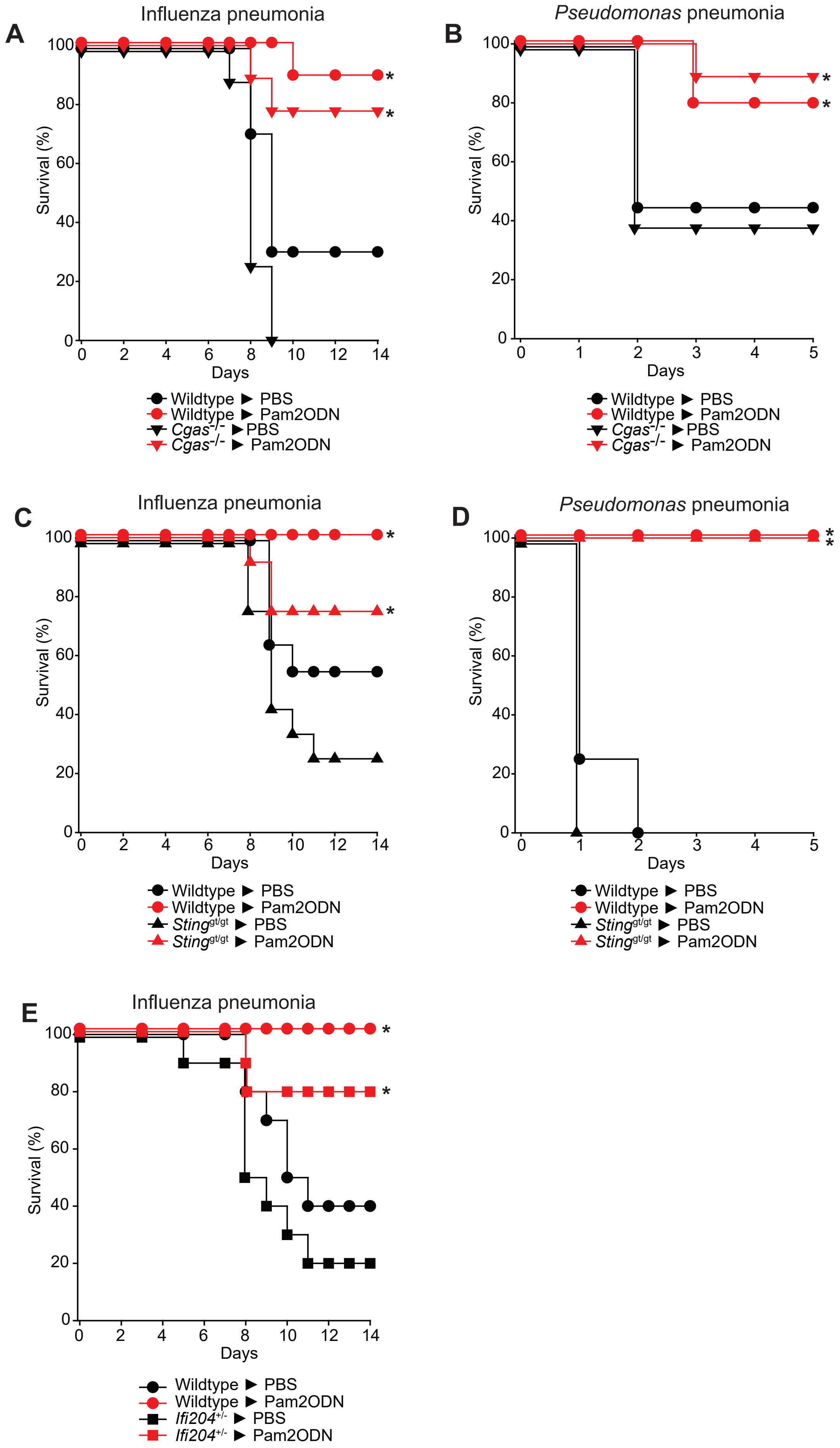

Wang, Supplemental Figure S2

A

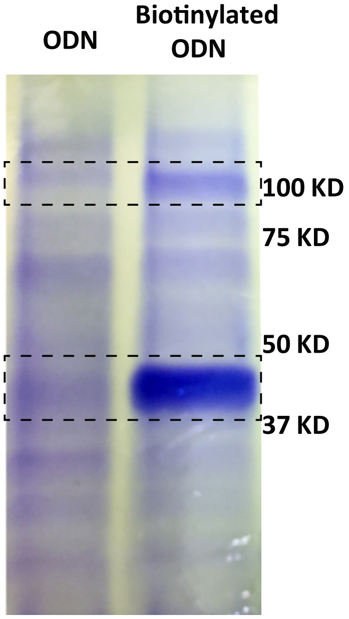

B

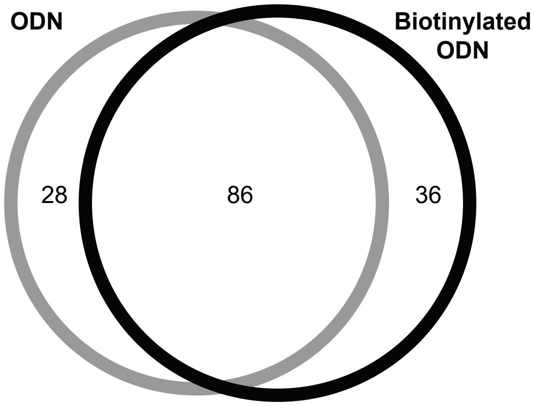

Wang, Supplemental Figure S3

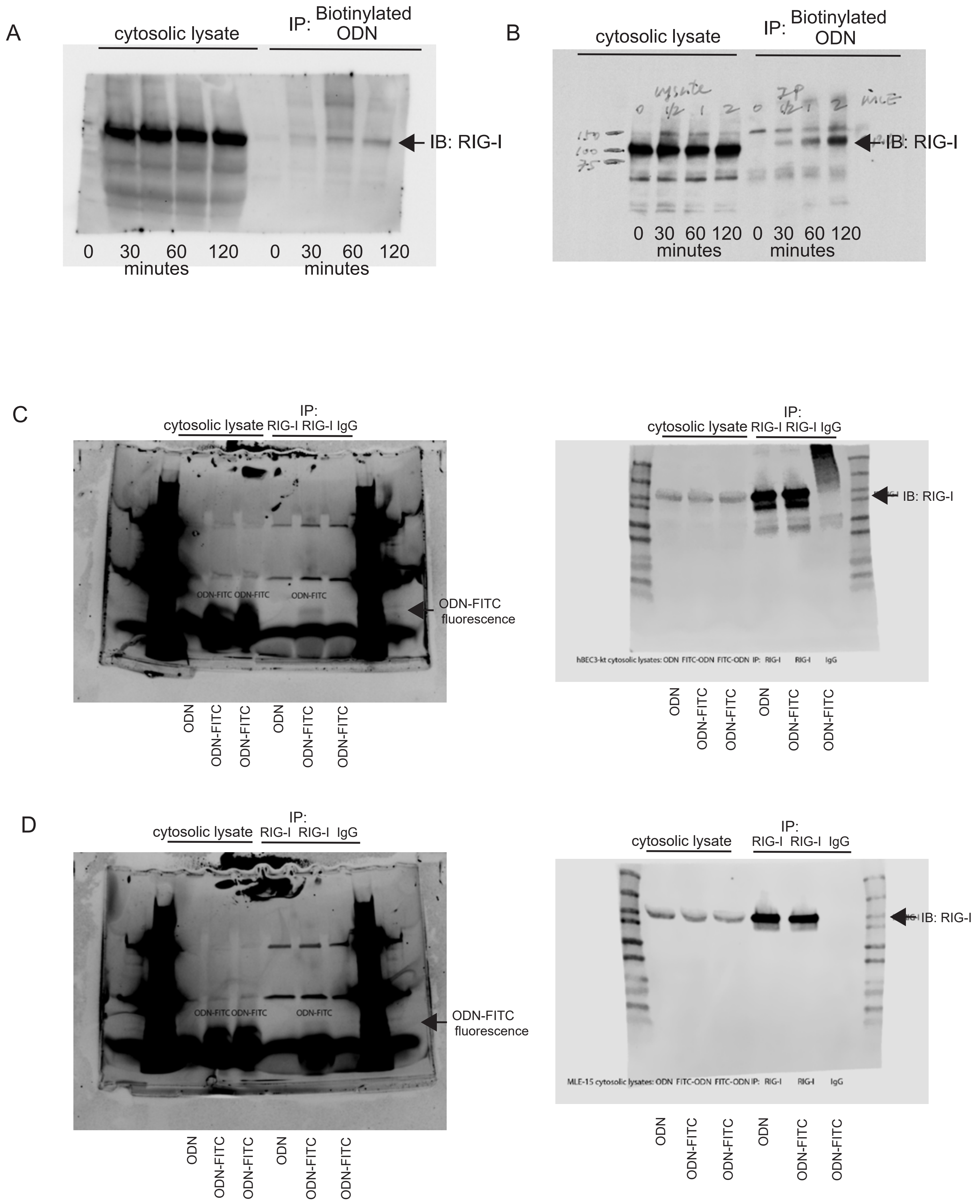

Wang, Supplemental Figure S4

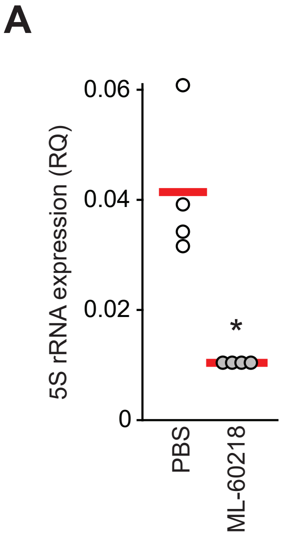

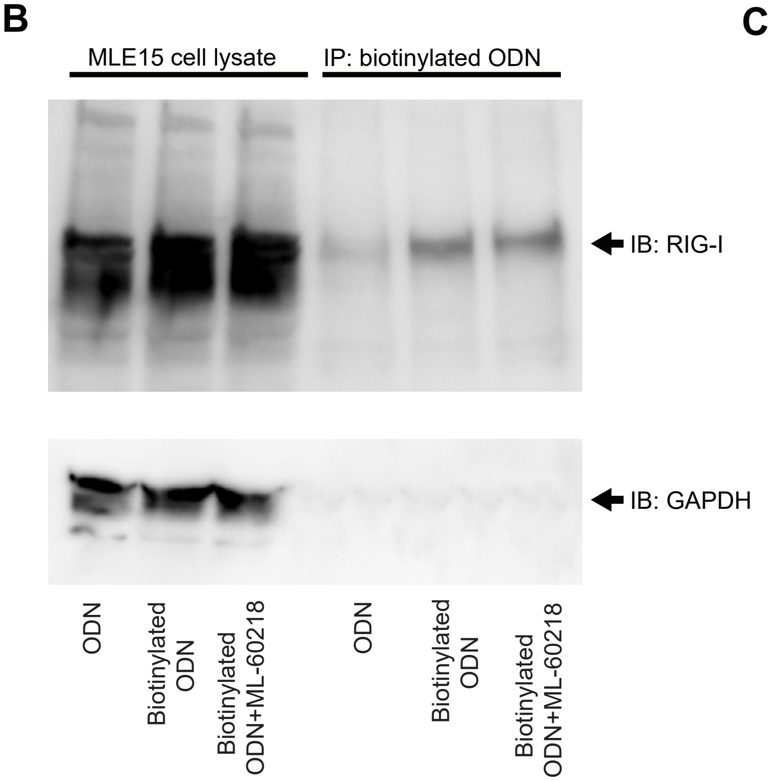

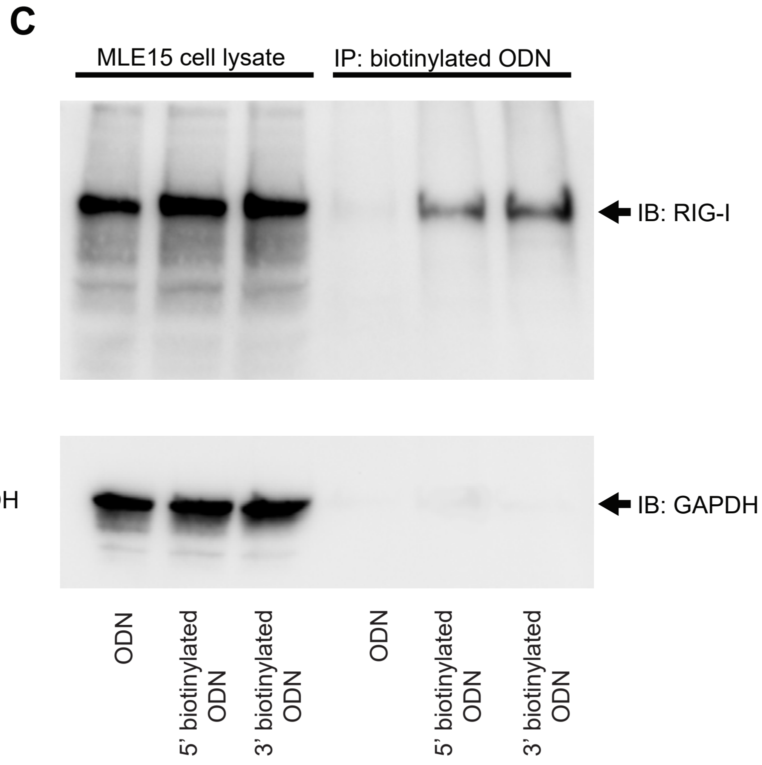

Wang, Supplemental Figure S5

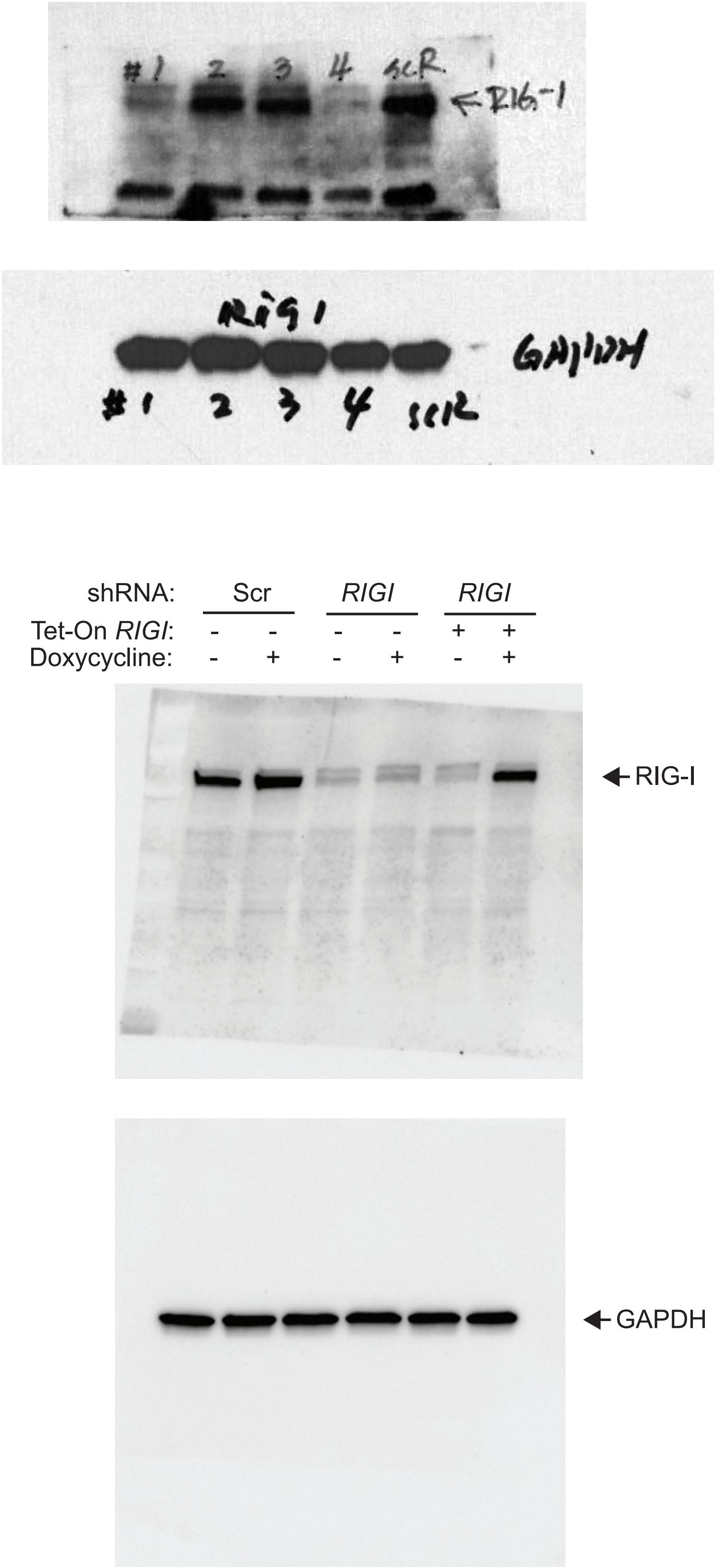

Wang, Supplemental Figure S6

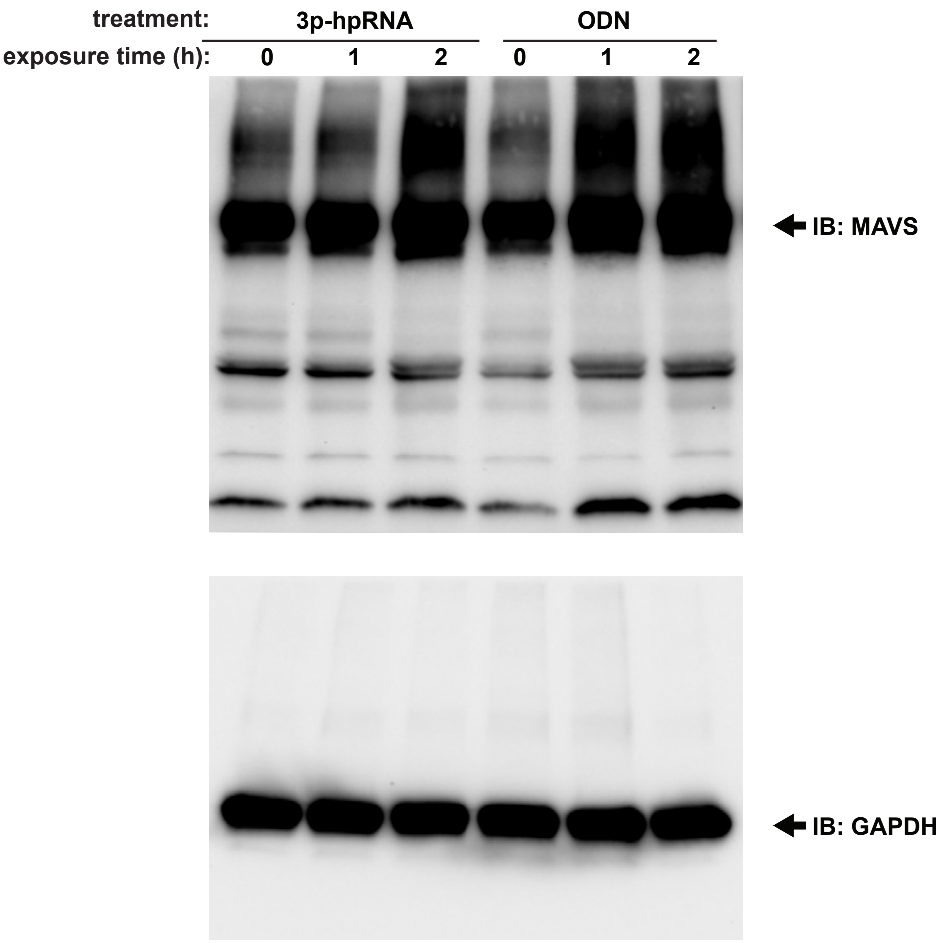

Wang, Supplemental Figure S7

A

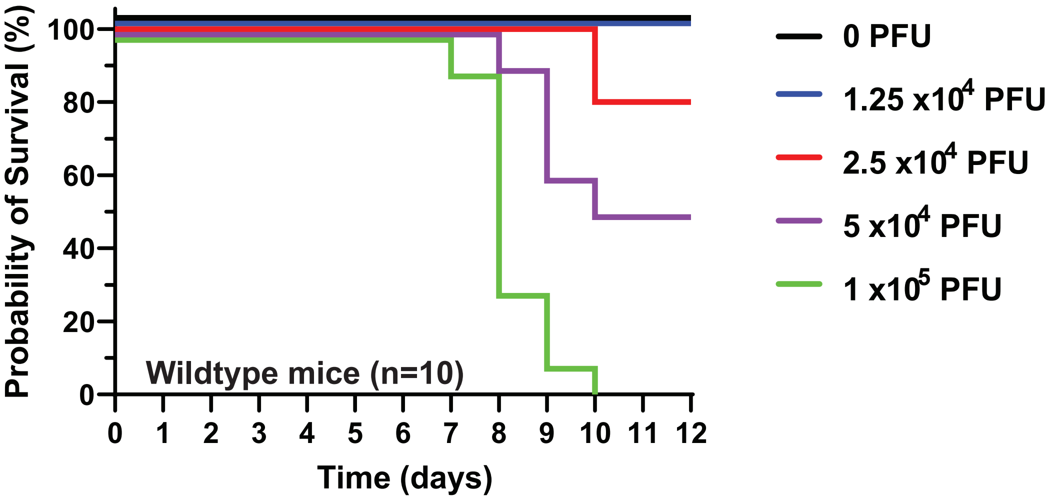

B

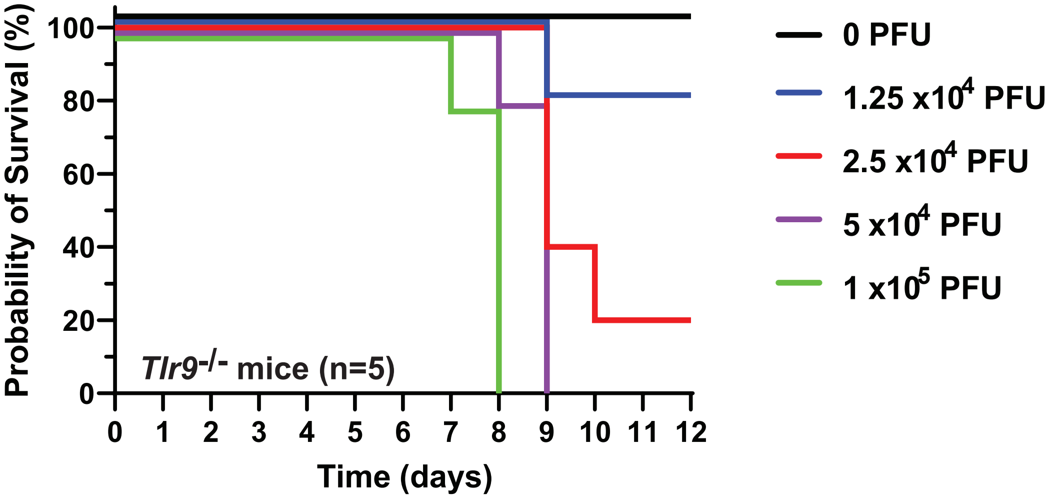

C

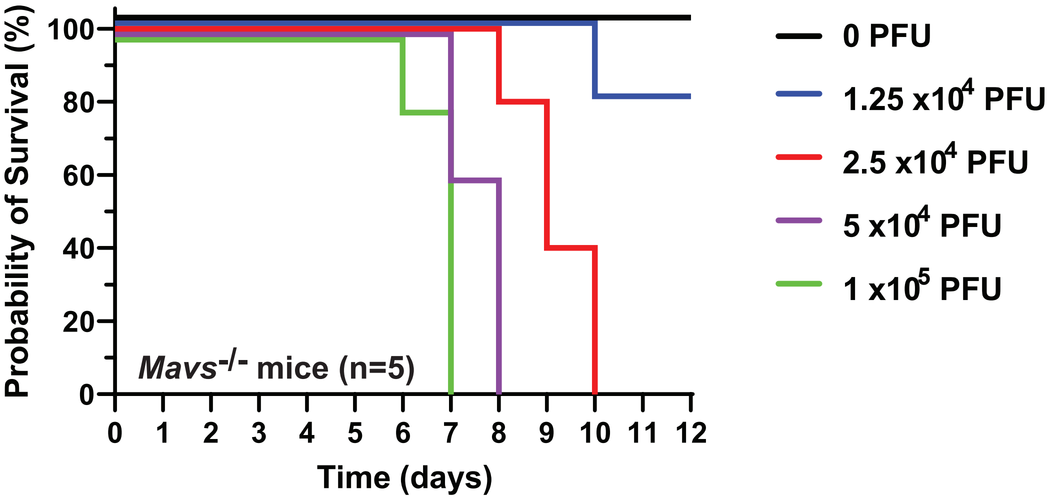

Wang, Supplemental Figure S8

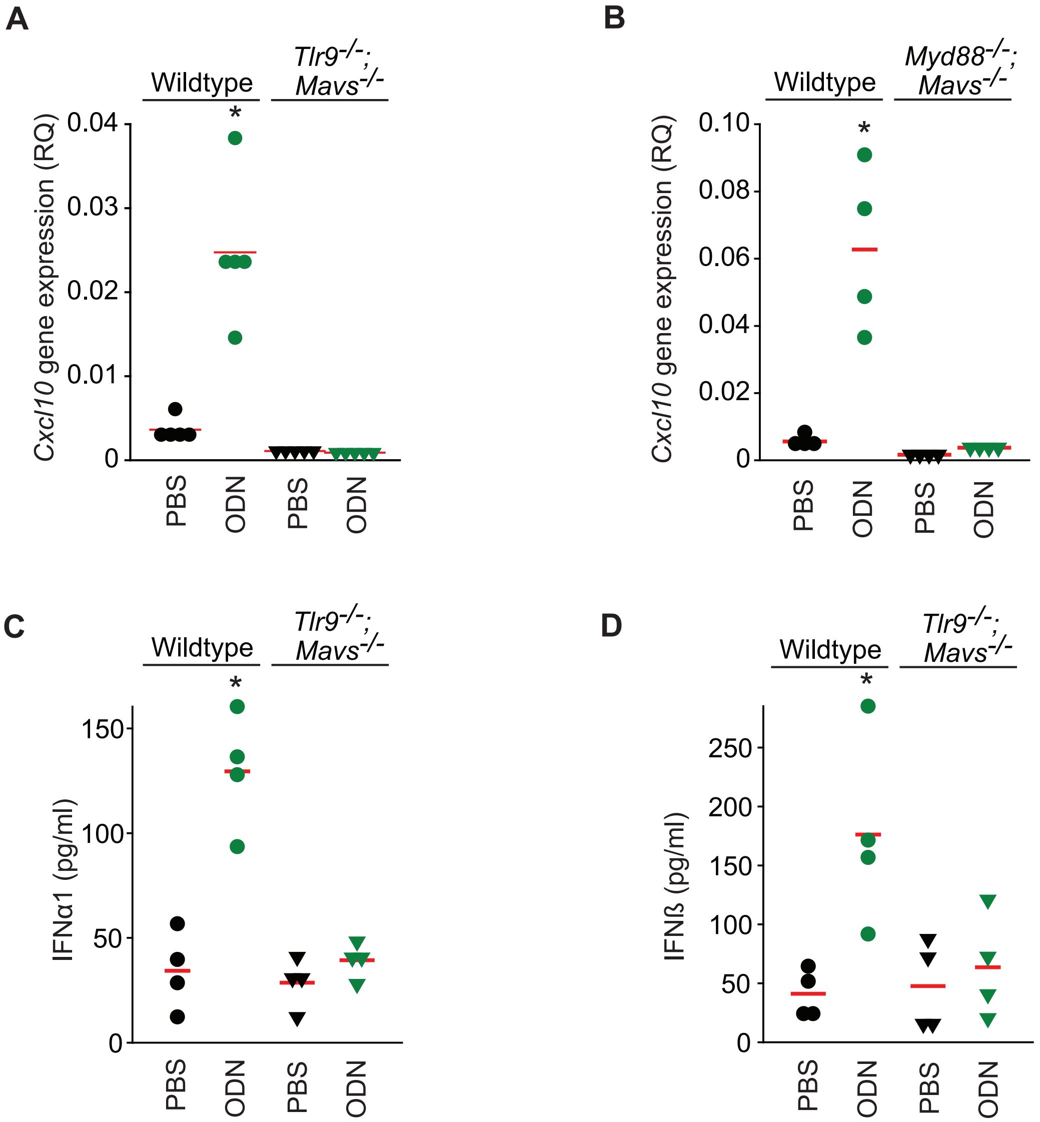

Wang, Supplemental Figure S9

A

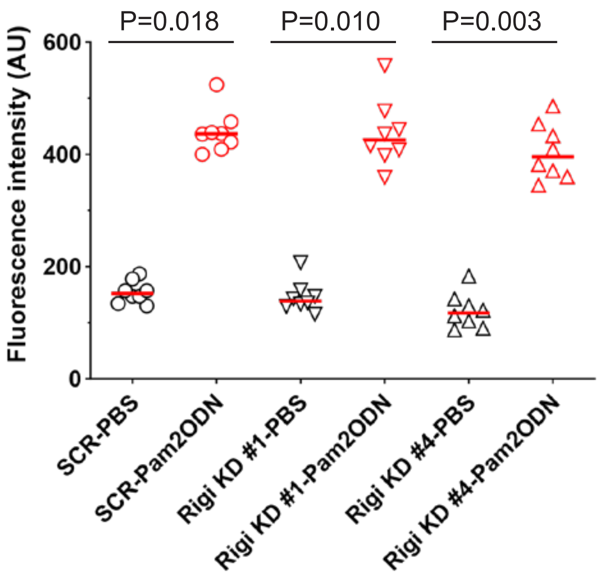

B

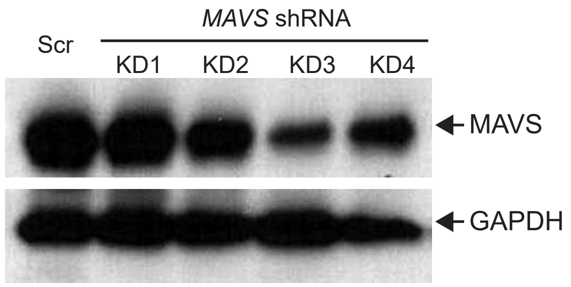

C

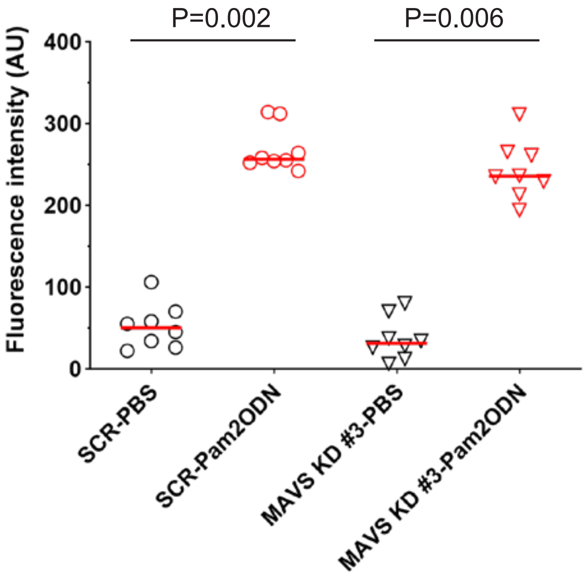

Supplement: Supplemental data [file jciinsight-9-180584-s153.pdf]
