# Supplementary material for: The RNA receptor RIG-I binding synthetic oligodeoxynucleotide promotes pneumonia survival
Source: JCI Insight. 2024 Nov 8;9(21):e180584. doi: 10.1172/jci.insight.180584 (PMC11601584; doi:10.1172/jci.insight.180584)

Wang, unedited blot, Figure 2A

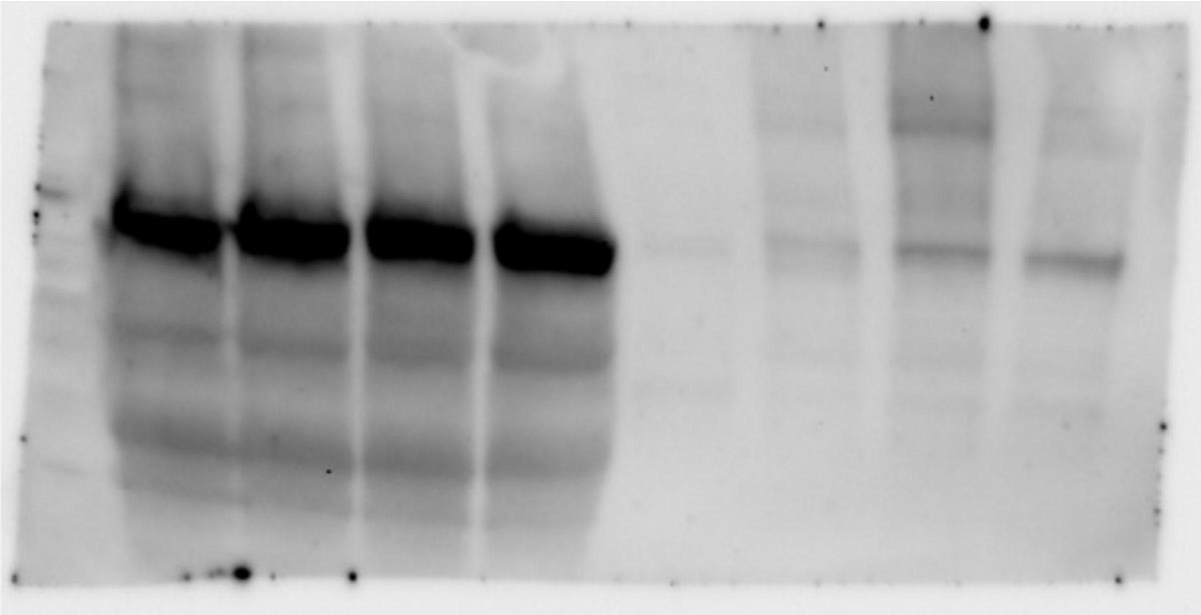

Wang, unedited blot, Figure 2B

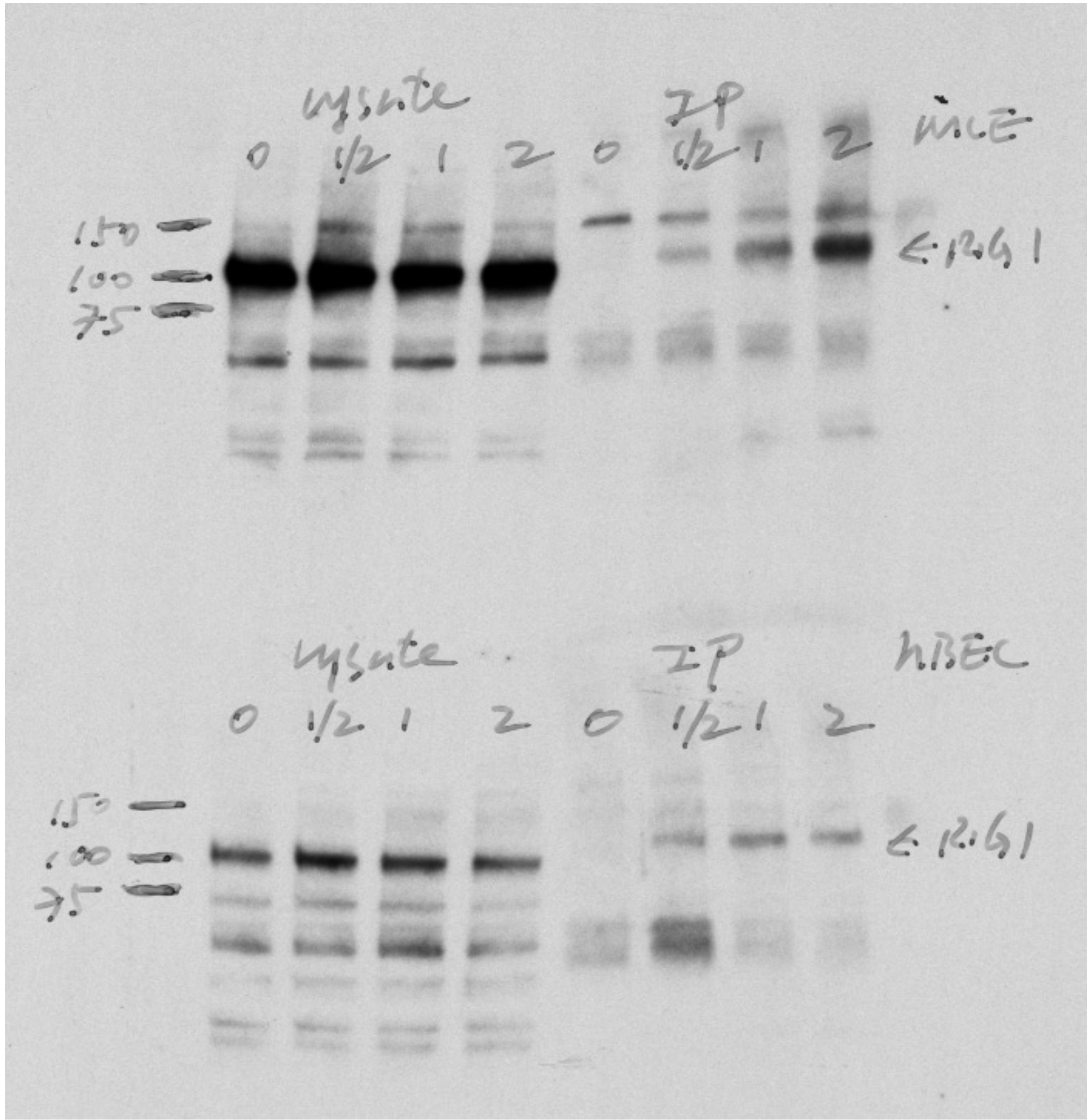

Wang, unedited blot, Figure 2C

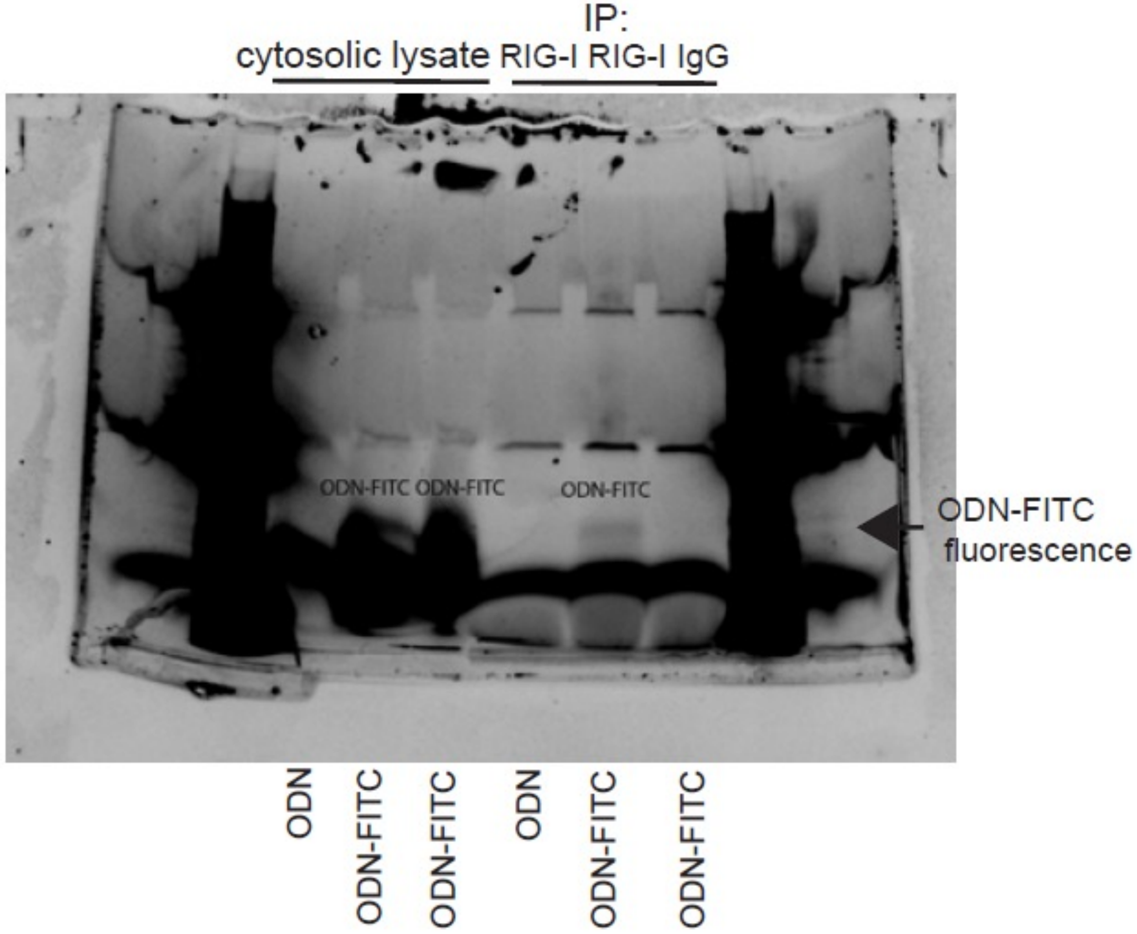

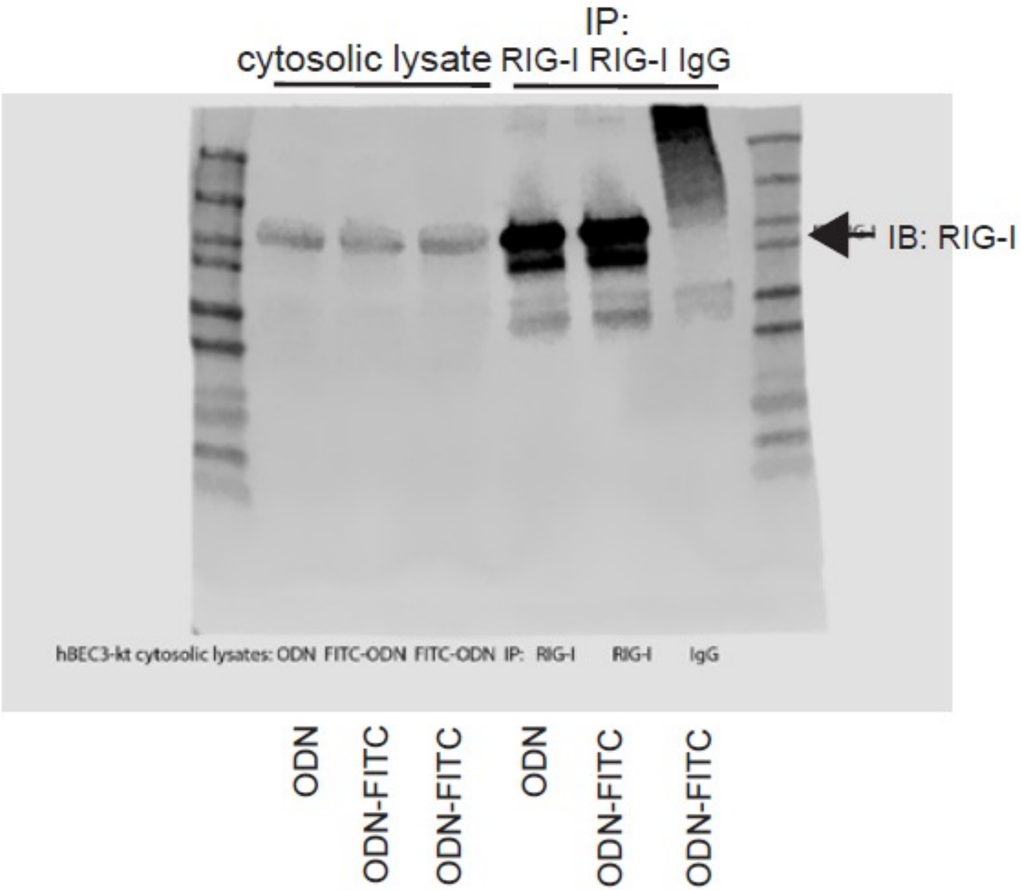

Wang, unedited blot, Figure 2D

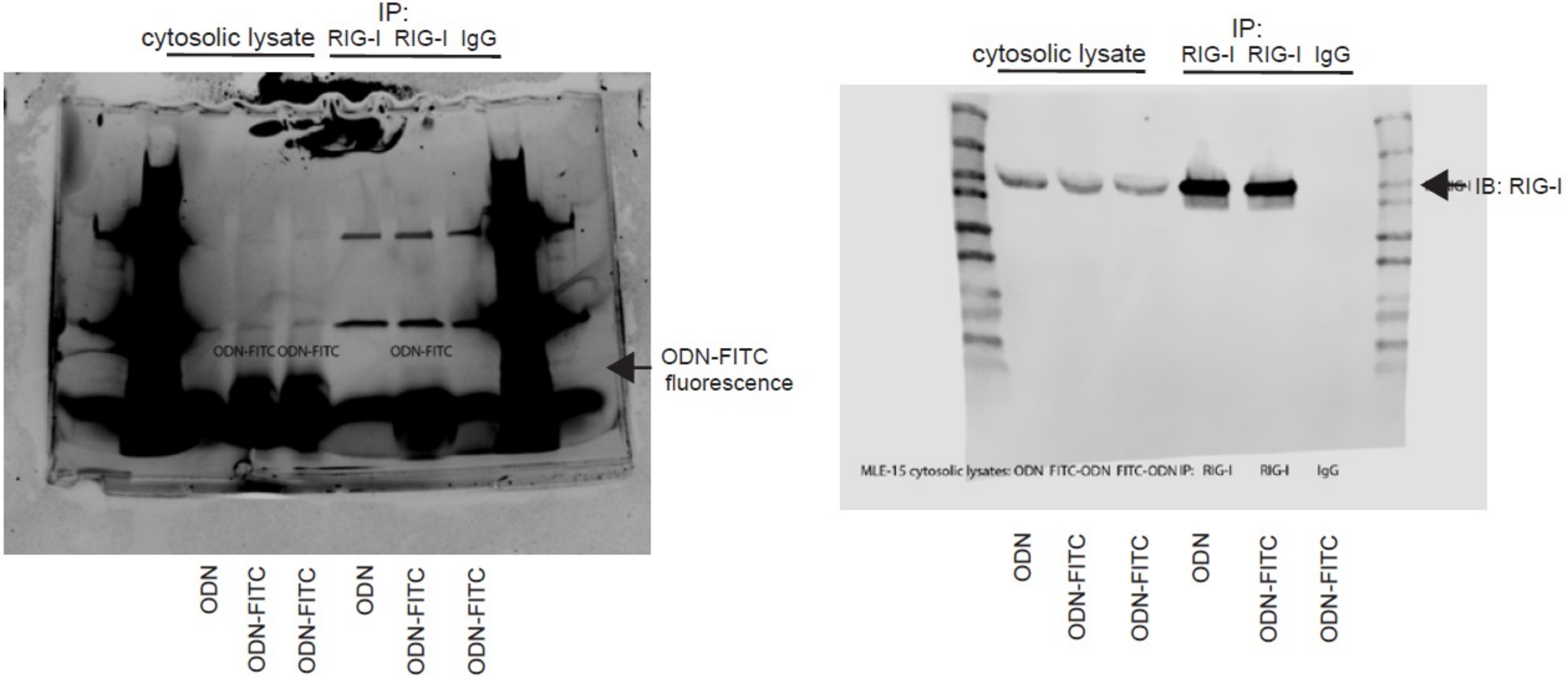

Wang, unedited blot, Figure 2E

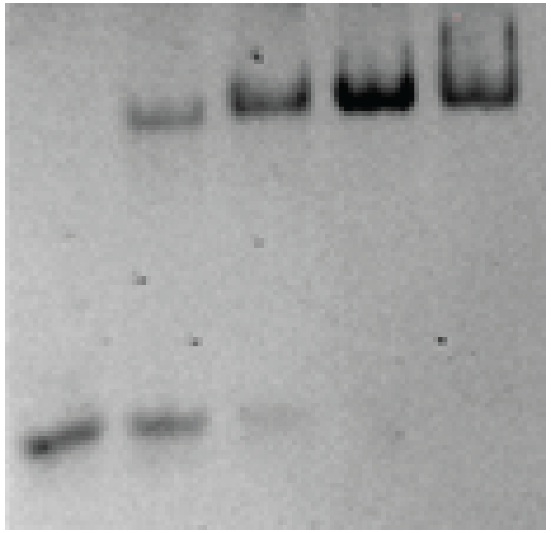

Wang, unedited blot, Figure 4A

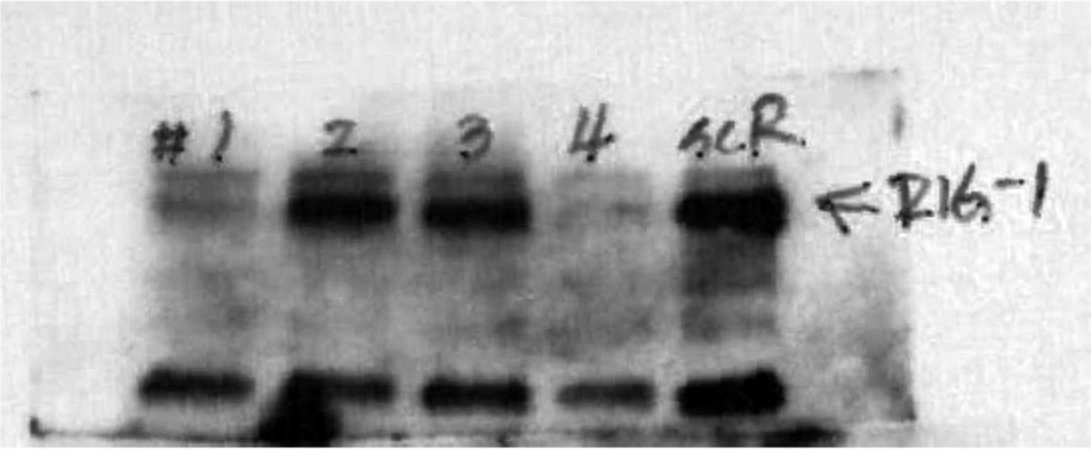

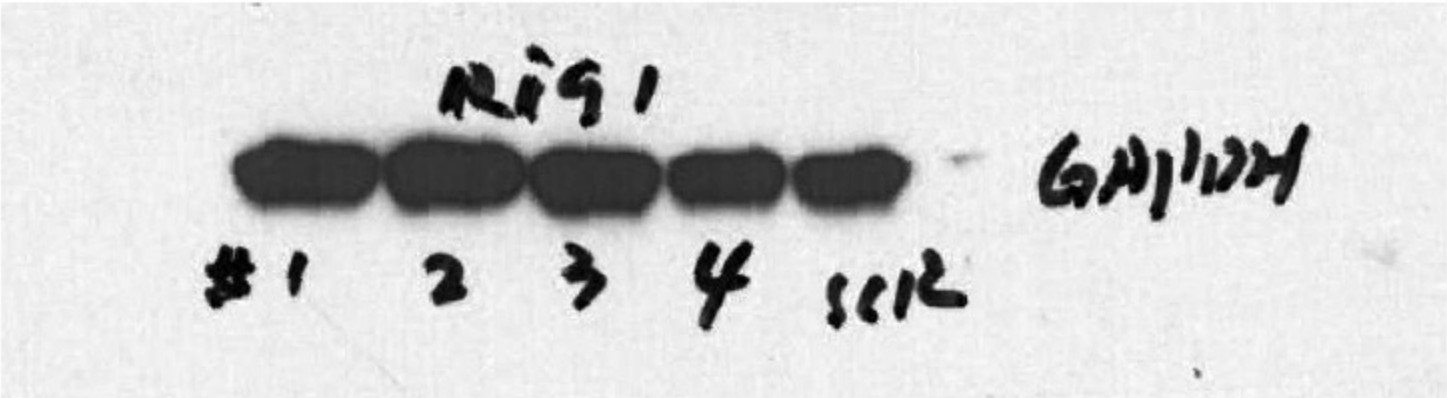

Wang, unedited blot, Figure 4F

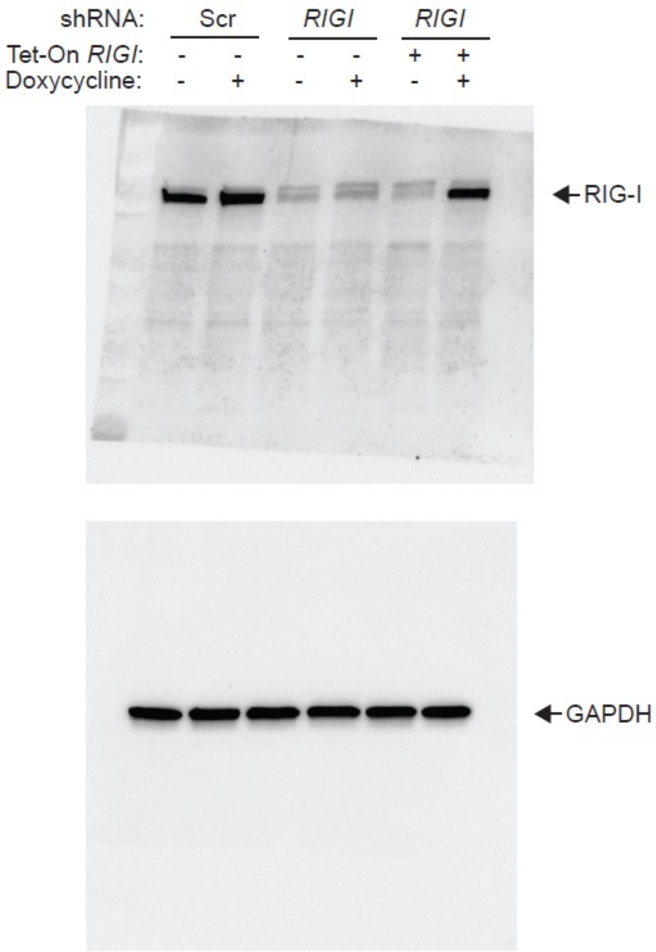

Wang, unedited blot, Figure S2A

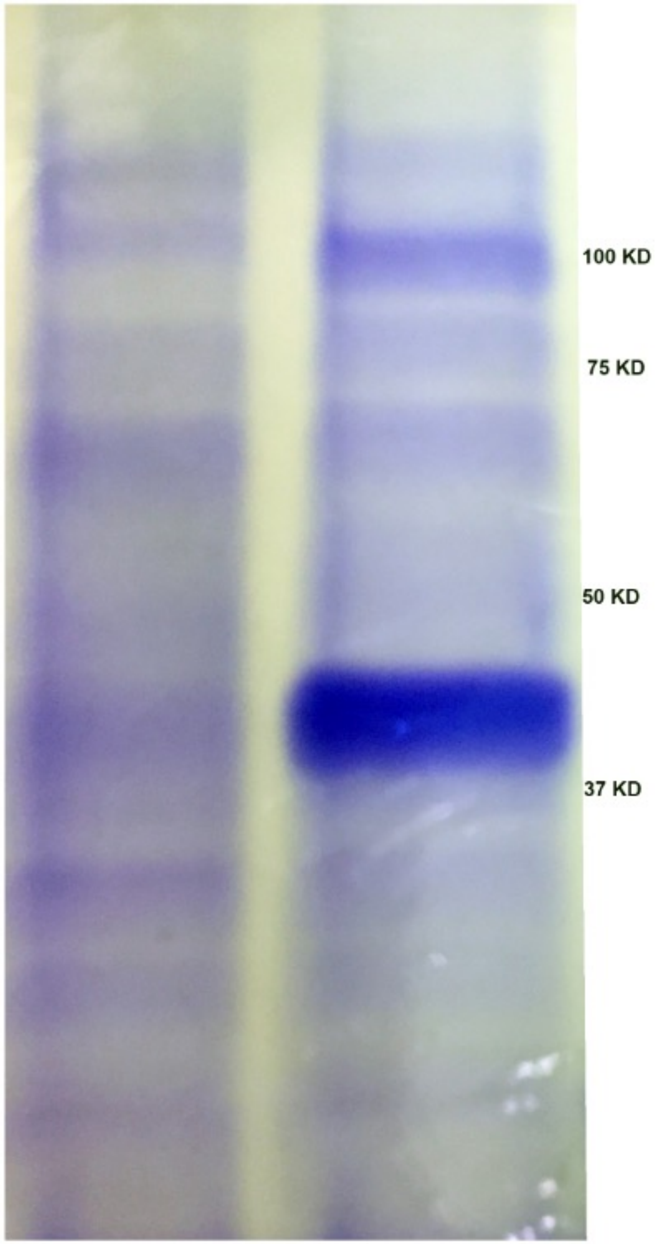

Wang, unedited blot, Figure S3B (GAPDH)

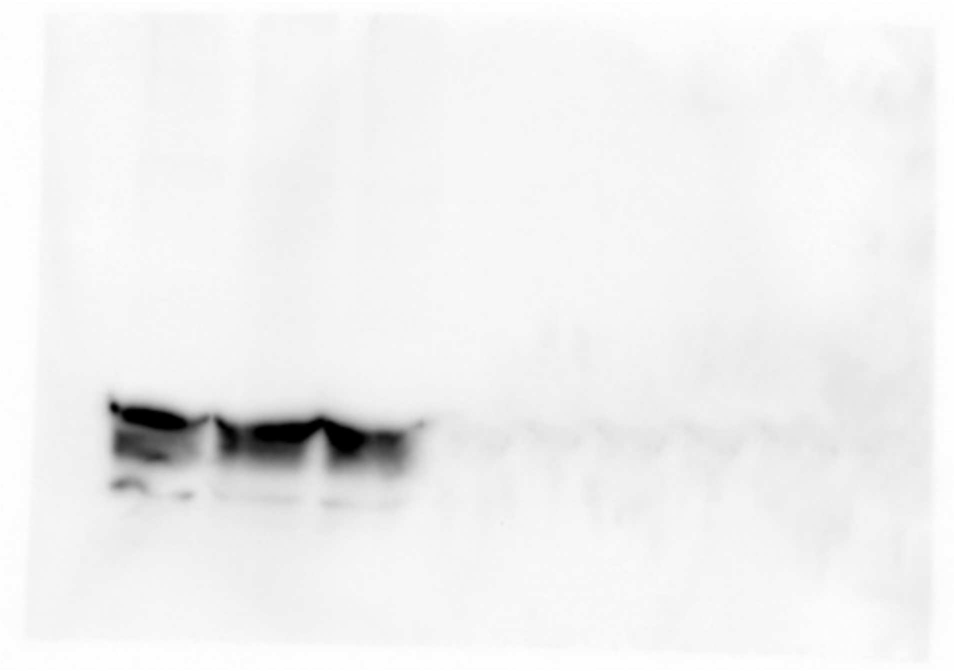

Wang, unedited blot, Figure S3B (RIG-I)

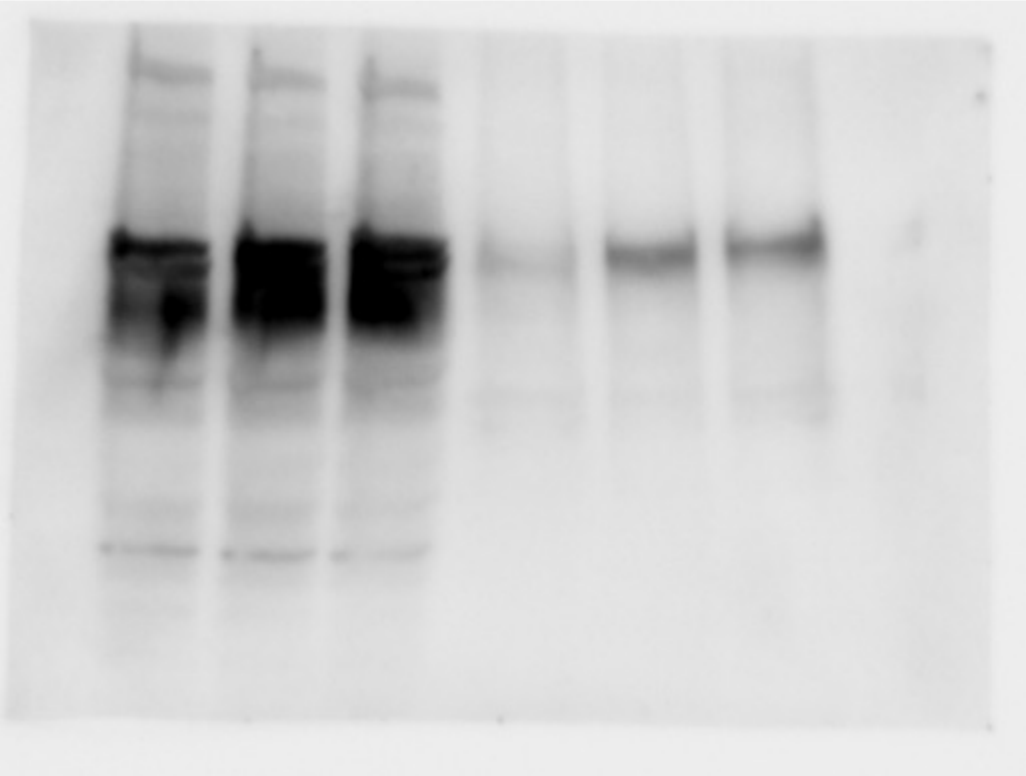

Wang, unedited blot, Figure S3C (GAPDH)

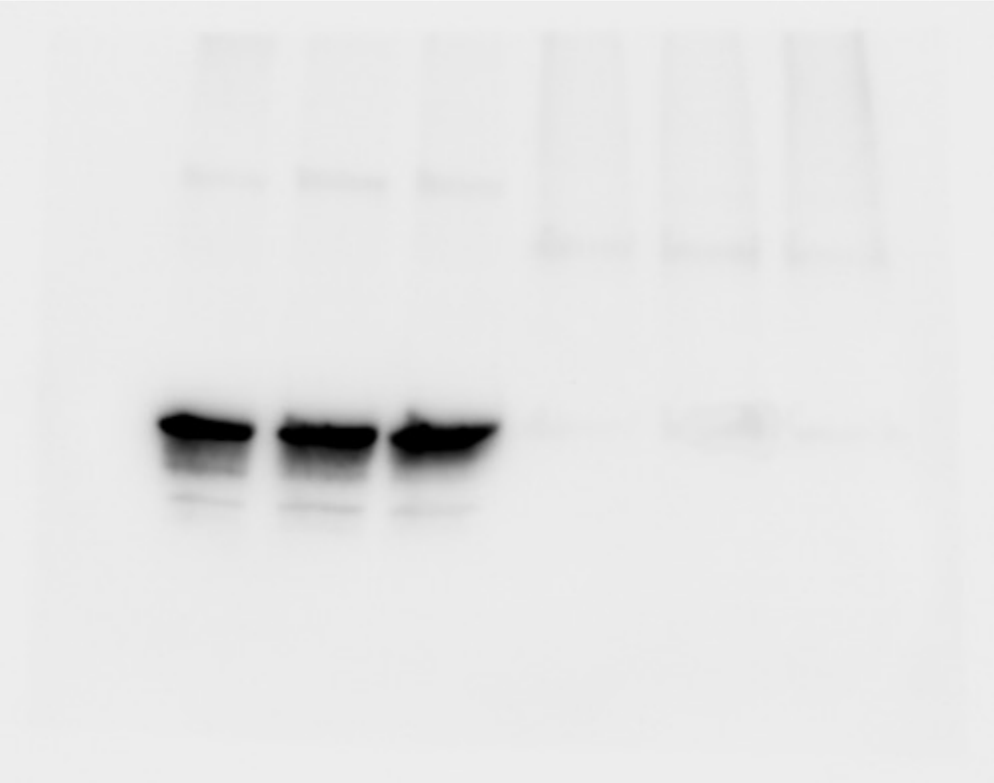

Wang, unedited blot, Figure S3C (RIG-I)

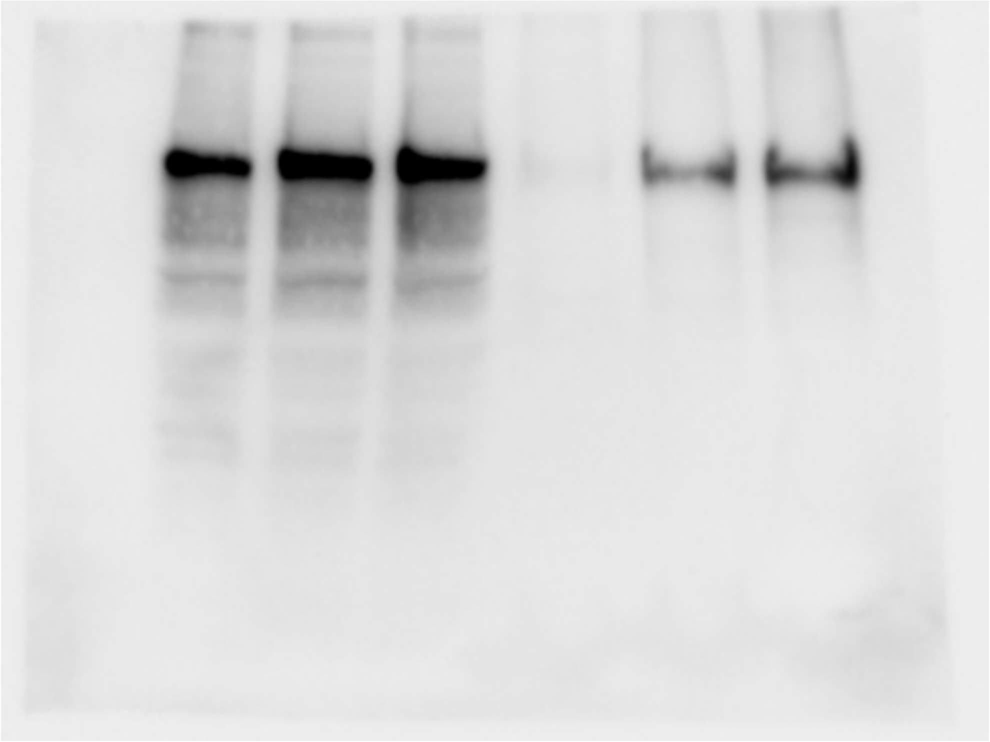

Wang, unedited blot, Figure S4A (GAPDH)

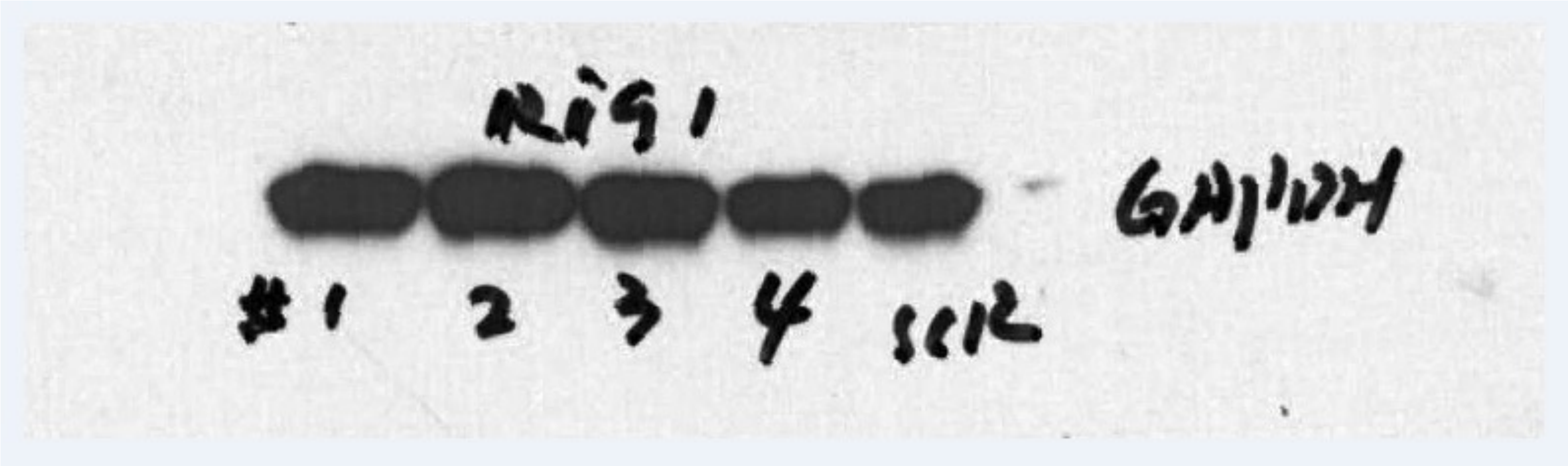

Wang, unedited blot, Figure S4A (RIG-I)

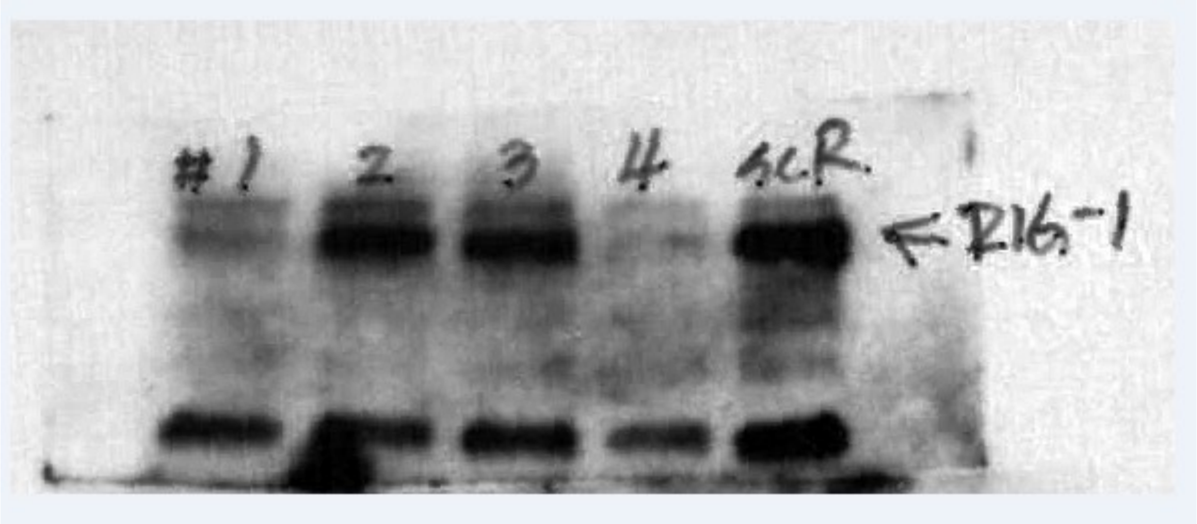

Wang, unedited blot, Figure S6 (GAPDH)

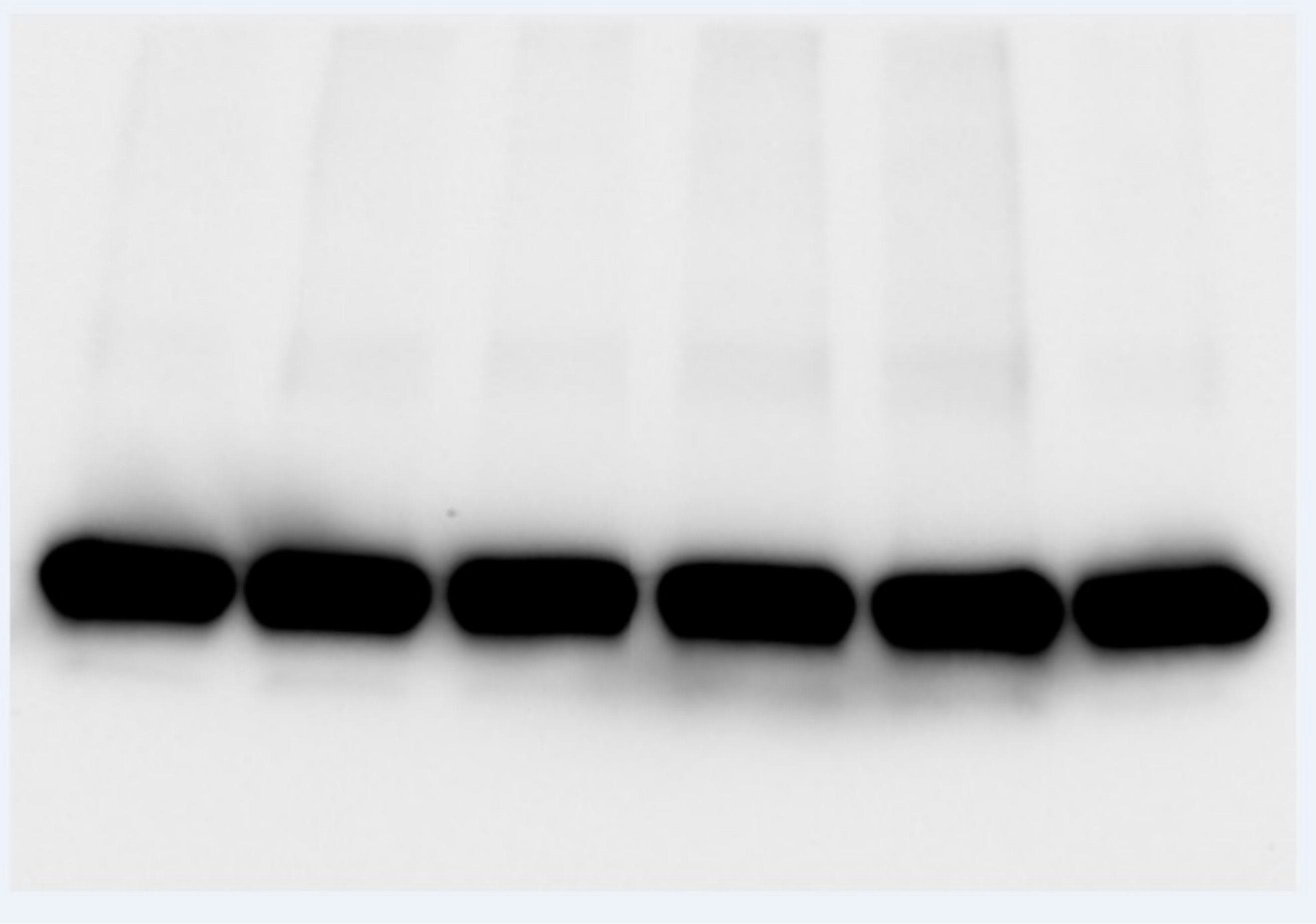

Wang, unedited blot, Figure S6 (MAVS)

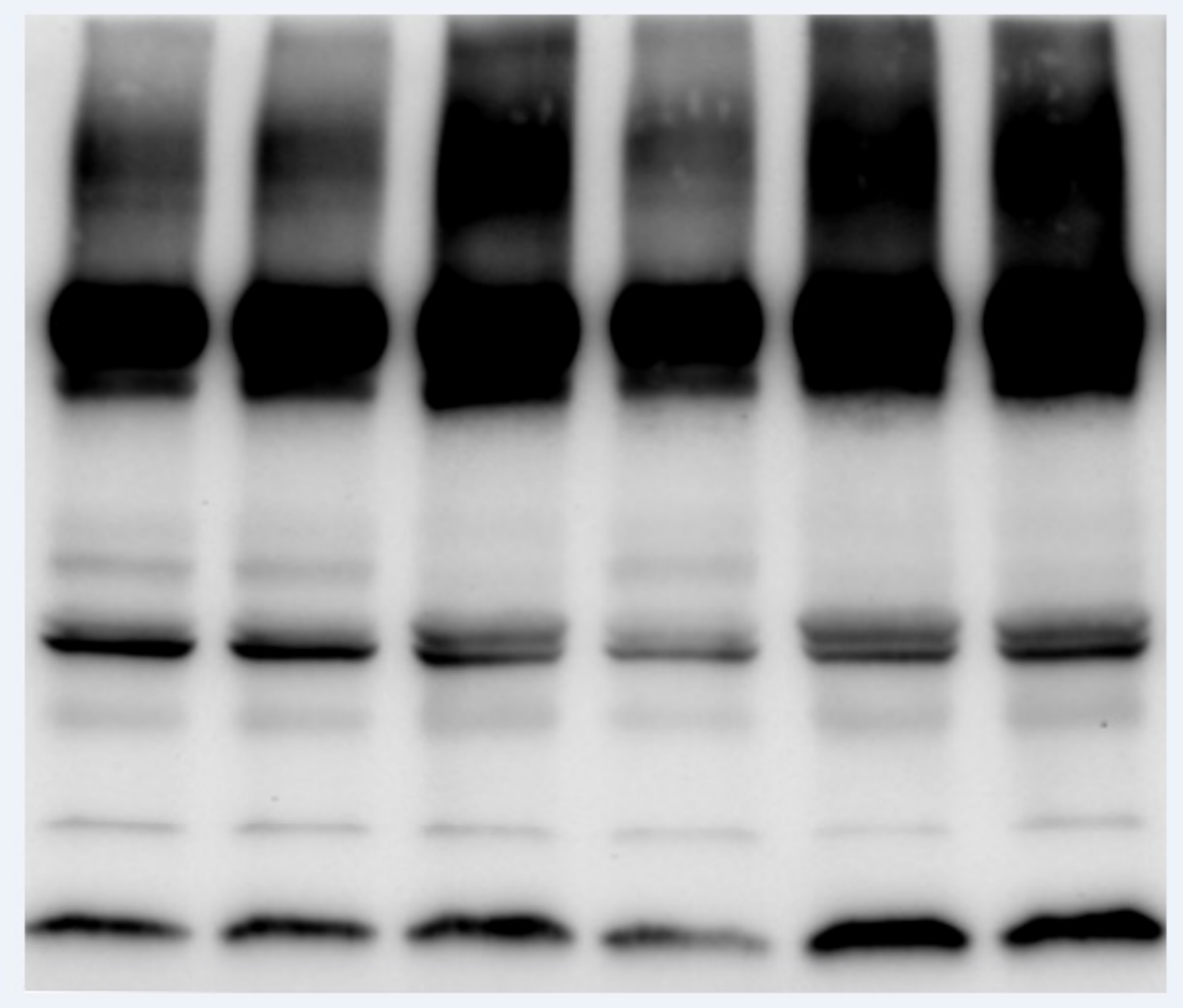

Wang, unedited blot, Figure S9B (GAPDH)

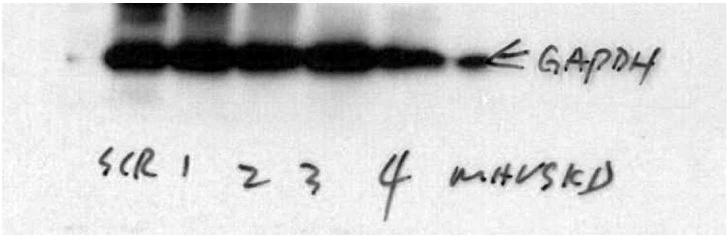

Wang, unedited blot, Figure S9B (MAVS)

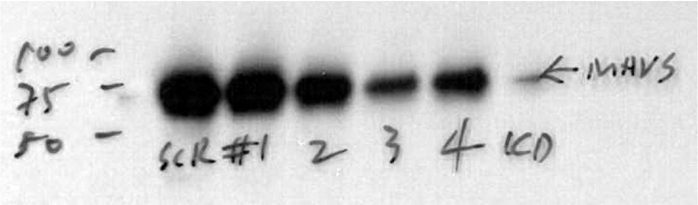

Supplement: Unedited blot and gel images [file jciinsight-9-180584-s154.pdf]
